# Understanding the performance of geographic limits on Web of Science Core Collection databases, using the United Kingdom as an example

**DOI:** 10.5195/jmla.2024.1669

**Published:** 2024-10-01

**Authors:** Helen A. Fulbright, Claire Stansfield

**Affiliations:** 1 helen.fulbright@york.ac.uk, Information Specialist / Research Fellow in Information Science, Centre for Reviews and Dissemination, University of York, York, United Kingdom; 2 c.stansfield@ucl.ac.uk, Senior Research Fellow, Evidence for Policy and Practice Information Centre (EPPI Centre), UCL Social Research Institute, University College London, London, United Kingdom

**Keywords:** Geographic search filters, information retrieval, literature searching, web of science, core collection

## Abstract

**Objective:**

To consider the approaches within Web of Science Core Collection (WoSCC) databases for limiting geographically. To compare the limits to an adaptation of NICE’s UK MEDLINE filter for use on WoSCC databases.

**Methods:**

We tested and appraised the inbuilt functions and search field options that support identification by countries/regions and affiliations. We compared these with an adapted filter to identify healthcare research on or about the UK. We calculated the recall of the inbuilt limits and filter using 177 studies and investigated why records were missed. We also calculated the percentage reduction of the overall number-needed-to-screen (ONNS).

**Results:**

Inbuilt limits within WoSCC enable identification of research from specific countries/regions or affiliations if there is data in the address field. Refining by affiliations allows retrieval of research where affiliations are in the 200 or 500 most frequent for a set of results. An adaptation of the UK MEDLINE filter achieved an average of 97% recall. ONNS was significantly reduced using the filter. However, studies where the countries or regions are only mentioned within the full text or other non-searchable fields will be missed.

**Conclusion:**

Information specialists should consider how inbuilt geographic limits operate on WoSCC and whether these are suitable for their research. The adapted filter can sensitively limit to the UK and could be useful for systematic reviews due to its high recall and ability to significantly reduce ONNS. Geographic filters can be feasible to adapt for use on WoSCC databases (where similar search fields are used between platforms).

## INTRODUCTION

Geographic limits or search filters aim to retrieve research with a common geographic location and are useful tools to focus the results of database searches [1]. Although many databases contain inbuilt limits, our experience is that there is often limited detail at the point-of-use on how these have been designed. Information specialists may not have time to investigate whether these are appropriate to use or could exclude relevant material. In contrast, user-developed search filters are more transparent, and often supported by data on information retrieval performance.

Searchers may have various reasons for wanting to restrict geographically: ranging from identifying research with a particular geographic focus or about specific populations, or to identify research undertaken by authors from certain regions. The searcher therefore needs to be aware of whether inbuilt limits or search filters have been designed to find research *on* or *from* a country (or both). Ayiku and colleagues suggest that the risk of excluding relevant material through untested methods may discourage the application of geographic restrictions and therefore increase the screening burden [2].

Data referencing geographic locations can be contained across a variety of search fields. For example, a United Kingdom (UK) filter for finding studies on or about the UK within MEDLINE (via Ovid), searches across the following fields: subject headings; title; abstract; journal word; institution; and country of publication [3]. However, geographic details may not be included in titles, abstracts, or subject headings (or not applied reliably), which affects the ability to find research on or about a given population [4]. Geographic-specific data might only be available at full text level, which is often non-searchable on database platforms [5]. Most geographic search filters are designed for use on MEDLINE or Embase [6, 7]. Only one geographic search filter, designed to retrieve research published by nursing scientists affiliated to German-speaking countries within specific nursing journals, has been published for the Web of Science Core Collection databases [8].

The Web of Science (WoS) platform is a valuable source of research papers and citations across a range of subjects [9, 10]. It facilitates bibliometric analyses (due to its origins as a citation index) and is also useful for systematic searches in health and social care [11]. The WoS platform should not be referred to as a single database, as it consists of several databases and database collections, depending on the subscription [12]. Users can search the databases of the Web of Science Core Collection (WoSCC) either individually or together. Given the large volume of content within this database collection, limiting by geographical location could be useful to focus search results. There are two inbuilt functions that refine the search results geographically (Countries/Regions or Affiliations). It would be useful to understand how they have been designed; what they are suitable for; and how reliable they are to use. It also prompts consideration of how these inbuilt limits compare with a geographic search filter adapted for use on WoSCC databases.

## OBJECTIVE

This paper aims to inform users about the performance of limiting geographically when searching for healthcare information on WoSCC databases. It describes a study exploring how the inbuilt limits (Countries/Regions and Affiliations) have been designed. It also tests and compares the performance of the inbuilt limits versus a translated search filter for retrieval of records on the UK.

## METHODS

Four processes were undertaken as follows:

1. Communication with WoS on the design of the inbuilt limits (Countries/Regions and Affiliations).
2. Adaptation of the UK MEDLINE filter to the WoSCC databases.
3. Testing the recall of the inbuilt limits and adapted search filter using four datasets on/about the UK.
4. Determining the reduction in overall number-needed-to-screen (ONNS) offered by the inbuilt limits versus the adapted search filter.

### 1. Understanding the Design of the Inbuilt Limits in WoSCC Databases (Countries/Regions and Affiliations)

Throughout 2022 and 2023, e-mail enquiries were made with the Web of Science Group (WoSG) support team on refining by Countries/Regions or by Affiliations on WoSCC databases. The enquiries asked which fields the WoSCC databases use to find data on Countries/Regions or Affiliations; whether affiliation data is combined with country (to differentiate between institutions with the same or similar names in different countries); and whether the names of countries, regions and affiliations is consistent or variable. WoSG were also asked whether the limits restrict to a certain number of results (and whether this could be increased), and if data is taken from all authors or only the lead author. The database help guides on refining results and viewing ‘Affiliation-Enhanced’ data also informed how the limits would perform and helped assess the strengths and limitations of each approach [13, 14].

### 2. Adapting the UK MEDLINE Filter to the WoSCC Databases

Ayiku et al.'s UK MEDLINE filter is a validated geographic search filter designed to retrieve research *on* or *about* the United Kingdom on Ovid MEDLINE with high recall and precision but will also retrieve research *from* the UK [15]. As Ovid MEDLINE and WoSCC databases have different search fields and there are no controlled subject headings on WoSCC, the adapted filter aimed to match the search syntax and search terms of the MEDLINE filter closely and make use of the available search fields in WoS. The search fields used in the adapted filter for WoSCC were:

- TS= terms in either title, abstract, author keywords, and keywords plus fields;
- TI= terms in title field;
- AB= abstract field;
- AD= address field (which will find institution and place names);
- CU= country/region in the address field;
- SO= publication titles field;
- OO= organization field; and
- OG= affiliation field (previously called organization-enhanced).

The process of adapting the MEDLINE filter involved checking the results retrieved from individual search lines to understand the search operations on WoS and then refining the filter accordingly. See the supplementary material for the filter and its line-by-line comparison to NICE's UK MEDLINE filter.

### 3. Testing the Recall of the Inbuilt Limits and Search Filter on Datasets on/about the UK

Four datasets were used to test the recall of the inbuilt limits and the adapted search filter. Datasets 1–3 consisted of 81 records on UK-based populations taken from three systematic reviews conducted by the EPPI Centre at University College London [16, 17, 18]. The full texts of these papers had been manually assessed and coded as being on a UK population, and they had not been limited to the UK in the searching or title and abstract screening stages of the review. Only records found on WoSCC databases were included in this dataset. Their presence in WoSCC was checked by selecting the database(s) used originally and searching the title field with suitable phrases.

Dataset 4 consisted of digital object identifiers (DOIs) that the National Institute for Health and Care Excellence (NICE) used as one of their gold-standard sets (GS3) for external validation of their UK MEDLINE filter. This contained references to research on or about UK populations that had been identified from the geographic setting of each paper. These are noted within the evidence description sections which summarize the included papers supporting NICE guidelines [19]. The DOIs were searched across the following six WoSCC databases and identified 96 records in this database collection:

- Science Citation Index Expanded (SCI-Expanded) 1900-present;
- Social Sciences Citation Index (SSCI) 1956-present;
- Arts & Humanities Citation Index (AHCI) 1975-present;
- Conference Proceedings Citation Index - Science (CPCI-S) 1990-present;
- Conference Proceedings Citation Index - Social Science & Humanities (CPCI-SSH) 1990-present; and
- Emerging Sources Citation Index (ESCI) 2015-present.

Once the 81 records for datasets 1–3 and the 96 records for dataset 4 were retrieved, the inbuilt geographic limits and translated filter were applied to investigate how many records were retained and why records were missed. For both inbuilt limits, the default option was used to refine the results, which lists the top 200 countries/regions or affiliations (i.e., the most frequent) within a set of results. The datasets are described in Table 1.

**Table 1:** Datasets Used

	**81 records consisting of:**
**Datasets 1–3**	6 records on SSCI taken from a review of children’s views about obesity, body size, shape and weight (dataset 1).
	14 records on SSCI, taken from a review on transition of young people from children's to adults' health and social care services (dataset 2).
	61 records on SSCI, CPCI-SSH and ESCI, taken from a review of public health service provision by community pharmacies (dataset 3).
**Dataset 4**	96 records on SCI-Expanded; SSCI; AHCI; CPCI-S; CPCI-SSH; and ESCI.

**Figure 1:**
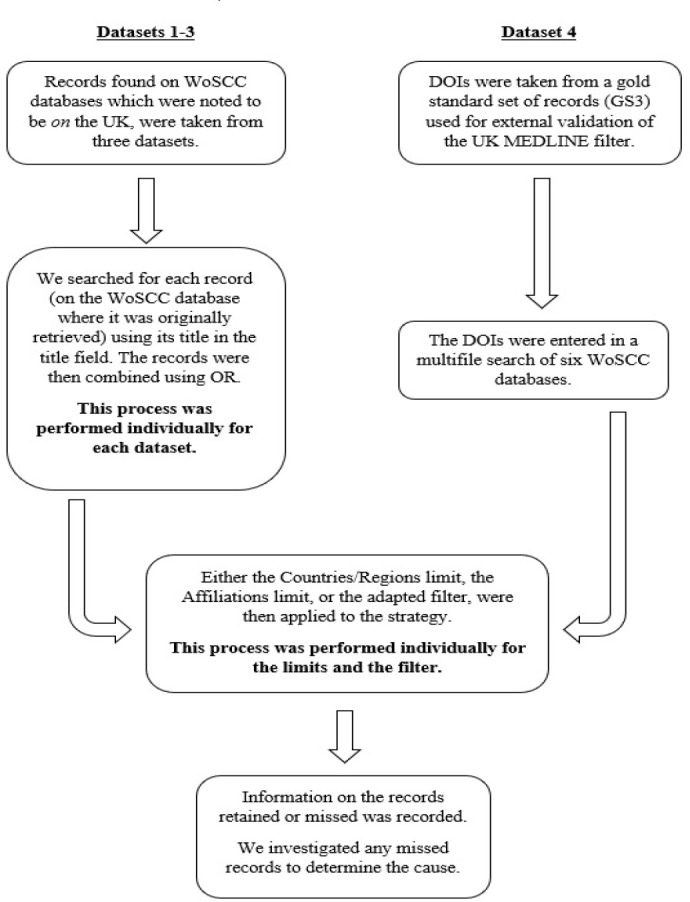
Testing the Recall of the Inbuilt Limits and Search Filter on Datasets on/about the UK

The overall process of testing the recall of the inbuilt limits and search filter is summarized in Figure 1. Full strategies are available in the supplementary material.

### 4. Determining the Reduction in ONNS offered by the Inbuilt Limits Versus the Adapted Search Filter:

ONNS is a term that refers to the overall number of records that need to be screened on title and abstract and is different from the typical number-needed-to-screen metric (which indicates the number of records needed to screen on title and abstract to find one included reference) [20]. Determining the reduction in ONNS therefore measures the reduced overall number of records that need to be screened. It is valuable to explore how ONNS is reduced when applying the inbuilt limits and filter as this shows the reduced screening burden.

The original search strategies from datasets 1 and 3 were replicated as closely as possible and then the inbuilt limits or translated UK filter were applied. Application of the Countries/Regions and Affiliations limit refined the results using the list of the top 200 countries/regions or affiliations within a set of results. It was expected that the reproduction searches would not retrieve the same number of results as the original searches, due to various factors such as updates to the search functionality of the database collection over time. For dataset 1, the original search retrieved 1,915 records, whereas 2,416 were retrieved by the reproduced search. For dataset 3, the original search retrieved 5,284 records, whereas 5,377 were retrieved by the reproduced search.

Reduction in ONNS was calculated by comparing the percentage change in the number of records retrieved when applying the inbuilt limits and filter to the reproductions of the original search strategies used for datasets 1 and 3. Datasets 2 and 4 could not be used, either due to changes to the search functionality of WoSCC databases affecting reproducibility (dataset 2), or due to no search strategy to reproduce (dataset 4).

## RESULTS

### 1. Using the Inbuilt Limits in WoSCC Databases (Countries/Regions and Affiliations)

The inbuilt limit for Countries/Regions is one of the options under ‘refine results’ and is designed to find or exclude research *from* a country rather than *on* a country. The limit uses the CU field (Countries/Regions), which is determined by the countries and regions listed for every author in the address field. When using this limit, the following UK choices may be listed: England, North Ireland, Scotland, Wales, UK and United Kingdom [21]. The country names are consistent, but their availability will vary depending on whether results contain author addresses from that country or region. It is not possible to select anything narrower such as individual towns or cities using this inbuilt limit.

The inbuilt Affiliations limit is designed to find or exclude research *from* an affiliation and uses the OG (Affiliation) field to search for all author affiliations. There are several points to note. Firstly, the OG field retrieves records containing an affiliation's preferred name or its name variants and is the only field that can find ‘Affiliation-Enhanced’ data [22, 23]. As an example, the University of Leeds is also referred to by the name variants ‘White Rose University Consortium’ and ‘N8 Research Partnership’ in the Affiliation-Enhanced data. Secondly, it is not always obvious which affiliation a name variant belongs to without further examination of the record's metadata (e.g., ‘Hospital for Sick Children SickKids’). Thirdly, for institutions that have campuses based in other countries the affiliation is listed as the main campus, and the country/region is listed as the actual location. For example, records by Oxford University Clinical Research Unit in Vietnam will show in results for Oxford University using the Affiliations limit (as well as its name variants) and in Vietnam and for the Countries/Regions limit. Fourthly, the OG field is still referred to by its previous name ‘Organization-Enhanced’ in WoS guides that have not been updated [24].

Expanding the Countries/Regions or Affiliations sections displays the top 200 countries/regions or affiliations (i.e., the most frequent) that are directly applicable to the user's results. The search bar allows the option to find countries/regions or affiliations applicable to the results, regardless of whether they are in the top 200 record count. Users can refine by up to a maximum of the 500 most frequent countries/regions or affiliations by selecting ‘Analyze Results’ and clicking on the ‘Web of Science Categories’ drop-down box to select either ‘Countries/Regions’ or ‘Affiliations’ [25]. This page shows the number of times each of the countries/regions or affiliations appears in a set of results (and how many records do not contain data in the address field). The minimum record count can be adjusted on this page to specify how many times countries/regions or affiliations must occur in the results, but the user must press enter to apply this before refining their results. There is also an option to obtain data on up to 100,000 frequent countries/regions or affiliations relevant to a set of results [26, 27]. However, there is no option to include records that do not have any data for the authors’ countries/regions or affiliations [28, 29].

Clarivate's further information about the Countries/Regions and Affiliations limits are contained in a section on advanced search field tags (for fields CU and OG, respectively) [30]. Additional information on the Affiliations limit can also be found on Clarivate's viewing affiliation-enhanced name(s) page [31].

### 2. Adapting the UK MEDLINE Filter to the WoSCC Databases

In adapting the UK MEDLINE filter, checking the results retrieved from individual search lines on WoSCC databases led to the following findings which influenced the adaptation of the filter. Firstly, Northern Ireland is abbreviated as North Ireland on WoSCC databases. Therefore, it was necessary to include the terms “North Ireland*” and “North Irish*”. Secondly, search terms for the National Health Service (NHS) retrieved results from non-UK National Health Services (e.g., Italy) with no UK authors. To reduce this noise, these terms were combined with UK country terms in the AD (address) field using the Boolean operator AND. Thirdly, the UK MEDLINE filter's use of the Boolean operator NOT for certain UK place names that might pick up irrelevant results (e.g., York NOT New York) could exclude records where researchers based in these locations have collaborated unless these records are found with other lines in the search filter. For this reason, in the adapted filter, certain UK place names were combined with relevant UK place names using the Boolean operator AND wherever these were searched for in the AD field.

It should be noted for these second and third points, where relevant UK place names were combined with certain search terms using the Boolean operator AND, many of these place names had been searched for elsewhere in the filter using different field tags. In comparison, the AD field searches for affiliation names as well as place names. See the supplementary material for further details on the adaptation of each line of the UK MEDLINE filter for use on WoSCC databases.

### 3. Testing the Recall of the Inbuilt Limits and Search Filter on Datasets on/about the UK

Recall is shown to vary in the datasets from the EPPI Centre (sets 1–3) and NICE (set 4). Tables 2 and 3 show how many of the records from datasets 1–3 and 4 were retained with each of the geographic limits applied and why records were missed.

**Table 2:** Recall for Datasets 1–3

Geographic Limit	Recall of Records (N=81)	Number of Missed Records	Missed Record Reason
UK Filter	81 (100%)	0 (0%)	N/A
Countries/Regions Limit	78 (96%)	3 (4%)	Non-UK affiliation (1) No data in address (2)
Affiliations Limit	77 (95%)	4 (5%)	Non-UK author address (1) No data in address (2) Ambiguous affiliation (1)

**Table 3:** Recall for Dataset 4

Geographic Limit	Recall of Records (N=96)	Number of Missed Records	Missed Record Reason
UK Filter	91 (95%)	5 (5%)	UK data in full text only (5)
Countries/Regions Limit	88 (92%)	8 (8%)	Non-UK author address (8)
Affiliations Limit	81 (84%)	15 (16%)	Ambiguous affiliation (1) Non-UK affiliation (9) Not in top 200 Affiliations (5)

### 4. Determining the Reduction in ONNS offered by the Inbuilt Limits versus the Adapted Search Filter

The original search strategies for datasets 1 and 3 were replicated as closely as possible. The replicated strategy for dataset 1 found 2,416 results, whereas the strategy for dataset 3 found 5,377 results. Table 4 shows how these figures were reduced with each geographic restriction applied.

**Table 4:** Reducing ONNS

	Dataset 1		Dataset 3	
Geographic Limit	2,416 records reduced to:	Reduction Amount	5,377 records reduced to:	Reduction Amount
UK Filter	631	1,785 (74%)	1,560	3,817 (71%)
Countries/Regions Limit	261	2,155 (89%)	698	4,679 (87%)
Affiliations Limit	168	2,248 (93%)	505	4,872 (91%)

## DISCUSSION

### Understanding and Using the Inbuilt Limits in WoSCC Databases (Countries/Regions and Affiliations)

For users to understand how WoSCC's inbuilt limits operate and how to use them effectively, information could be clearer at the point-of-use. For instance, the Countries/Regions limit could specify ‘Countries/Regions (of authors)’. The limits could also include a description that only the top 200 countries/regions or affiliations are displayed (as the default option) and that the search box can be used to find specific countries/regions or affiliations of interest [32].

Knowledge on how WoSCC's inbuilt limits have been designed (i.e., to search in the address field) helps users to understand what they might need to do to include results without data in the address field and enhance recall. For instance, an option to obtain records without data on countries/regions could be useful. This can be achieved by applying the limit to exclude all countries and regions from the results, repeating this step to include the countries and regions that *are* of interest, and then using the Advanced Search Query Builder to combine these two sets together using OR. Simply excluding *only* the irrelevant countries and regions would not achieve the same result. This is because authors from different countries or regions can work together, so their exclusion could inadvertently remove results of interest. Using a similar method with the Affiliations limit to obtain records without affiliation data is possible. However, as the method involves excluding all affiliations from the results this could require repeated iterations of search lines that exclude all affiliations, since there are so many affiliations and only a maximum of 500 can be excluded on a single search line.

For users looking to use their own search terms rather than relying on the inbuilt limits, searching using the OG (Affiliation) field tag could be a useful option to find affiliation names as well as name variants for affiliations, whereas the field tag OO (Organization) could be used where particular affiliation names are wanted [33]. Alternatively, it is possible to search the AD (Address) field which will search for affiliations or place names within a record's address field [34]. However, users should be aware that only the OG field will pick up Affiliation-Enhanced data.

### Comparing the Recall of the Inbuilt Limits and Search Filter on Datasets on/about the UK

The recall of the inbuilt limits and adapted search filter was consistently high because research on the UK was typically produced by UK authors. The recall of the UK filter was 100% for datasets 1–3 and 95% for dataset 4 (averaging 97%). In comparison, recall was slightly reduced using either of the inbuilt geographic limits for datasets 1–3 and 4. For Countries/Regions, recall was 96% and 92% respectively (averaging 94%) and for Affiliations 95% and 84% respectively (averaging 89%).

The high recall of the Countries/Regions limit supports its application as a precise way of geographically limiting to the UK or other countries when conducting literature searches, though this is not its intended purpose. However, in the context of other geographic locations, it is unclear whether studies on the country of investigation are typically conducted by researchers from that country, or whether this could prejudice the results toward certain research fields.

The recall of the Countries/Regions limit could be enhanced if there was an option to include records without data in the address field. However, this could increase noise (and records containing relevant geographic data outside of the address field would still be missed). For datasets 1–3, two of the three records missed by the Countries/Regions limit had no data in the address field. The same records were also missed by the Affiliations limit for this reason.

The Affiliations limit missed records for a variety of reasons, including records without an affiliation in the top 200 record count, ambiguous data, and non-UK affiliations. Overall, the main reason the Affiliations limit missed records was due to non-UK affiliations. For dataset 4, six of nine missed records mentioned the UK in the full text only; one mentioned the UK in the abstract; and the remaining two had errors in the metadata meaning UK affiliations were not listed. This highlights the importance of searching the abstract field to find records on a particular geographic location. It also demonstrates that records can be missed due to incorrect metadata in WoSCC databases.

An issue with the application of the Affiliations limit is its ambiguous data. For instance, the affiliation ‘New Croft CTR’ (representing the New Croft Centre in Newcastle, UK) isn't easily identifiable as a UK affiliation without further investigation and was the cause of one of the fifteen missed records for datasets 1–3.

The high recall of the adapted UK filter was due to its application of a broader range of search fields in comparison to the inbuilt limits. It can be important to search publication titles to retrieve research on or about a specific population. Notably, the filter's application of the SO (publication) field tag allowed it to find a record in the *British Journal of Clinical Psychology* by Australian authors which was missed by both inbuilt limits.

In total, only five records were missed by the filter. These were all multi-country studies, where UK data was only accessible in the full text. This is one of the main limitations of the UK filter (which also applies to the NICE UK MEDLINE filter). However, these multi-country studies were also missed by the inbuilt limits.

### Determining the Reduction in ONNS offered by the Inbuilt Limits Versus the Adapted Search Filter

The overall number of records retrieved was substantially reduced using either of the inbuilt limits or the adapted filter compared to the use of no geographic restrictions. However, this could be at the expense of retrieving relevant records, especially for sensitive searches designed to find records *on* rather than *from* a geographic location. The adapted filter offered a large percentage reduction in ONNS (an average of 72% fewer records) even though this was the lowest reduction overall due to its more sensitive performance versus the inbuilt limits.

The tests performed to show the reductions in ONNS aimed to show an *approximate* reduction. Records can change database within WoSCC over time. Clarivate note that records are indexed at journal-level and, depending on the journal performance, could move from ESCI to a flagship database such as SCI-Expanded, SSCI, or AHCI [35].

### Applying the Findings to Other Contexts

As most of the UK terms in the adapted filter are generic, it could be applied to UK searches conducted outside of a healthcare context if terms for the NHS were removed. Sutton and Campbell's study on adapting a lower middle-income country filter makes the point that ‘[p]erformance of filters can be varied and may need adapting to the needs of research topics' [36]. The adapted filter could be modified to incorporate geographic terms that reflect the context the filter is being used for, such as words for relevant regions, counties, towns, villages, or affiliations. A further consideration is that inbuilt language limits could help to limit geographically. Although language limits may not always be applied precisely (for example, English is used to disseminate work to an international readership), using other language limits on WoSCC could prove useful if trying to find records from populations where research is written in specific languages. For example, it may be useful to restrict to records written in Swedish, if retrieval of records about Sweden is a goal.

Geographic filters can be feasible to adapt for use with WoSCC databases (where similar search fields are used between platforms), and other filters could be developed in the same way. Although the focus of this paper has been on the UK, similar approaches could be used for other countries. For example, to identify research *on* or *about* low and middle-income (LMIC) countries, it would be important to use topic searches for the country in addition to the Countries/Regions limit to improve identification of relevant research. Using additional terms for LMICs would improve sensitivity of such a filter (e.g., see https://epoc.cochrane.org/lmic-filters).

The ‘Analyze Results’ feature available to use with WoSCC databases could be beneficial in the adaptation or creation of new geographic search filters to test and refine search filters as they are being developed. For each line of the search entered onto the database(s), relevant Web of Science Categories (e.g., Publication Titles, Languages, Countries/Regions etc) could be selected in the drop-down box for ‘Analyze Results’ which has downloadable data for up to 100,000 results.

## LIMITATIONS

This is an exploratory study based on data that were readily available to the authors and was conceived with literature searching in mind. It therefore may not fully reflect applications of the WoSCC inbuilt limits for conducting bibliometric studies that focus on where the research is produced. The inbuilt limits were not designed for locating studies that are *about* a geographical population, and inevitably they had lower performance than a filter that was designed to do so. The original searches for datasets 1 and 3 were conducted during 2008 and 2017 respectively and the volume of results would likely be considerably higher if updated to the current date. So far, the adapted filter has only been tested using four datasets on healthcare literature, comprising 177 records, which is 12% of the size tested for the NICE UK MEDLINE filter [37].

## CONCLUSION

It is beneficial to understand how or whether to use the WoSCC inbuilt geographic limits for different use cases. Many database platforms that have designed inbuilt limits lack information on how these operate or how to use them effectively, which could give a false sense of security to less experienced searchers or cause expert searchers to be wary of using them altogether. This study therefore increases the awareness of searchers and information specialists in their application of geographic limits on WoSCC databases. For healthcare systematic reviews, the adapted UK filter appears to be an effective method to retrieve data both *on* and *from* the UK, as geographic data can be represented across a range of search fields. The filter will pick up everything retrieved by the Countries/Regions limit and can significantly reduce ONNS, though there is scope for further enhancements. Although the focus is on the UK, the findings are informative for identifying research from or about other geographical areas, and for using geographic limits and search fields on WoSCC databases.

## Data Availability

Data associated with this article are available in the supplementary material.
