# Supplementary material for: Understanding the performance of geographic limits on Web of Science Core Collection databases, using the United Kingdom as an example
Source: J Med Libr Assoc. 2024 Oct 7;112(4):332–40. doi: 10.5195/jmla.2024.1669 (PMC11486044; doi:10.5195/jmla.2024.1669)
Supplement: Supplementary file 1 — Appendix A: Supplemental Materials [file jmla-112-4-332-s01.docx]

**Supplementary Material**

[**1.** **Adapting the UK MEDLINE Filter to the WoSCC Databases:** 1](#_Toc177470126)

[The Adapted Filter: 1](#_Toc177470127)

[The Adapted Filter Compared to the UK MEDLINE Filter: 3](#_Toc177470128)

[Changes Made to the Adapted Filter: 4](#_Toc177470129)

[**2.** **Testing the Recall of the Inbuilt Limits and Search Filter on Datasets on/about the UK (Datasets 1-3):** 5](#_Toc177470130)

[Dataset 1: 5](#_Toc177470131)

[Dataset 2: 7](#_Toc177470132)

[Dataset 3: 10](#_Toc177470133)

[Dataset 4: 14](#_Toc177470134)

[**3.** **Determining the Reduction in Overall Number-Needed-to-Screen offered by the Inbuilt Limits Versus the Adapted Search Filter:** 18](#_Toc177470135)

[Dataset 1: 18](#_Toc177470136)

[Dataset 3: 21](#_Toc177470137)

# **Adapting the UK MEDLINE Filter to the WoSCC Databases:**

The adapted filter is shown below in addition to a table showing the line-by-line process of adapting the original UK MEDLINE filter and a written description of changes.

The WoSCC filter is not an exact translation and was first created for use in the following systematic review but has undergone additional changes prior to this study of its performance:

Murphy PJ, Hinde S, Fulbright HA, Padgett L, Richardson G. Methods of assessing value for money of UK-based early childhood public health interventions: a systematic literature review, *British Medical Bulletin*. 2023;145(1):88–109. <https://doi.org/10.1093/bmb/ldac035>

## **The Adapted Filter:**

# 1 TS=("National Health Service*" or NHS*) or (OO=(National Health Service) or OG=(NHS*) or AD=(National Health Service or NHS*)) and (AD=(Britain* or UK or "United Kingdom*" or England* or "North Ireland*" or "Northern Ireland*" or Scotland* or Wales*))

# 2 TI=(English not ((published or publication* or translat* or written or language* or speak* or literature or citation*) NEAR/5 English)) or AB=(English not ((published or publication* or translat* or written or language* or speak* or literature or citation*) NEAR/5 English))

# 3 TS=(GB or Britain* or (British* not "British Columbia") or UK or "United Kingdom*" or (England* not "New England") or "Northern Ireland*" or "Northern Irish*" or "North Ireland*" or "North Irish*" or Scotland* or Scottish* or ((Wales or "South Wales") not "New South Wales") or Welsh*) or SO=(Britain* or British* or UK or "United Kingdom*" or England* or "Northern Ireland*" or "Northern Irish*" or Scotland* or Scottish* or Wales* or Welsh*) or CU=(Britain* or UK or "United Kingdom*" or England* or "North Ireland*" or "Northern Ireland*" or Scotland* or Wales*)

# 4 TI=(Bath* or (Birmingham* not Alabama*) or Bradford* or Brighton* or Bristol* or Carlisle* or (Cambridge* not (Massachusetts* or Boston* or Harvard*)) or (Canterbury* not Zealand*) or Chelmsford* or Chester* or Chichester* or Coventry* or Derby* or (Durham* not (Carolina* or NC)) or Ely* or Exeter* or Gloucester* or Hereford* or Hull* or Lancaster* or Leeds* or Leicester* or (Lincoln* not Nebraska*) or (Liverpool* not ("New South Wales*" or NSW)) or (London* not (Ontario* or ONT or Toronto*)) or Manchester* or (Newcastle* not ("New South Wales*" or NSW)) or Norwich* or Nottingham* or Oxford* or Peterborough* or Plymouth* or Portsmouth* or Preston* or Ripon* or Salford* or Salisbury* or Sheffield* or Southampton* or "St Alban*" or Stoke* or Sunderland* or Truro* or Wakefield* or Wells or Westminster* or Winchester* or Wolverhampton* or (Worcester* not (Massachusetts* or Boston* or Harvard*)) or (York* not ("New York*" or NY or Ontario* or ONT or Toronto*))) or AB=(Bath* or (Birmingham* not Alabama*) or Bradford* or Brighton* or Bristol* or Carlisle* or (Cambridge* not (Massachusetts* or Boston* or Harvard*)) or (Canterbury* not Zealand*) or Chelmsford* or Chester* or Chichester* or Coventry* or Derby* or (Durham* not (Carolina* or NC)) or Ely* or Exeter* or Gloucester* or Hereford* or Hull* or Lancaster* or Leeds* or Leicester* or (Lincoln* not Nebraska*) or (Liverpool* not ("New South Wales*" or NSW)) or (London* not (Ontario* or ONT or Toronto*)) or Manchester* or (Newcastle* not ("New South Wales*" or NSW)) or Norwich* or Nottingham* or Oxford* or Peterborough* or Plymouth* or Portsmouth* or Preston* or Ripon* or Salford* or Salisbury* or Sheffield* or Southampton* or "St Alban*" or Stoke* or Sunderland* or Truro* or Wakefield* or Wells or Westminster* or Winchester* or Wolverhampton* or (Worcester* not (Massachusetts* or Boston* or Harvard*)) or (York* not ("New York*" or NY or Ontario* or ONT or Toronto*))) or AD=(Bath* or (Birmingham* and (Midlands* or England* or UK or "United Kingdom*")) or Bradford* or Brighton* or Bristol* or Carlisle* or (Cambridge* and (Cambridgeshire* or England* or UK or "United Kingdom*")) or (Canterbury* and (Kent* or England* or UK or "United Kingdom*")) or Chelmsford* or Chester* or Chichester* or Coventry* or Derby* or (Durham* and ("County Durham*" or England* or UK or "United Kingdom*")) or Ely* or Exeter* or Gloucester* or Hereford* or Hull* or Lancaster* or Leeds* or Leicester* or (Lincoln* and (Lincolnshire* or England* or UK or "United Kingdom*")) or (Liverpool* and (Merseyside* or England* or UK or "United Kingdom*")) or (London* and (Borough* or Westminster* or "Kingston-upon-Thames" or Kensington* or Chelsea* or Greenwich* or England* or UK or "United Kingdom*")) or Manchester* or (Newcastle* and (Tyne* or Wear* or England* or UK or "United Kingdom*")) or Norwich* or Nottingham* or Oxford* or Peterborough* or Plymouth* or Portsmouth* or Preston* or Ripon* or Salford* or Salisbury* or Sheffield* or Southampton* or ("St Alban*" and (Hertfordshire* or England* or UK or "United Kingdom*")) or Stoke* or Sunderland* or Truro* or Wakefield* or Wells or Westminster* or Winchester* or Wolverhampton* or (Worcester* and (Worcestershire* or England* or UK or "United Kingdom*")) or (York* and (Yorkshire* or England* or UK or "United Kingdom*")))

# 5 TI=(Bangor* or Cardiff* or Newport* or "St Asaph*" or "St David*" or Swansea*) or AB=(Bangor* or Cardiff* or Newport* or "St Asaph*" or "St David*" or Swansea*) or AD=(Bangor* or Cardiff* or Newport* or "St Asaph*" or ("St David*" and (Pembrokeshire* or Wales* or UK or "United Kingdom*")) or Swansea*)

# 6 TI=(Aberdeen* or Dundee* or Edinburgh* or Glasgow* or Inverness or (Perth* not Australia*) or Stirling*) or AB=(Aberdeen* or Dundee* or Edinburgh* or Glasgow* or Inverness or (Perth* not Australia*) or Stirling*) or AD=(Aberdeen* or Dundee* or Edinburgh* or Glasgow* or Inverness or (Perth* and (Perthshire* or Scotland* or UK or "United Kingdom*")) or Stirling*)

# 7 TI=(Armagh* or Belfast* or Lisburn* or Londonderry* or Derry* or Newry*) or AB=(Armagh* or Belfast* or Lisburn* or Londonderry* or Derry* or Newry*) or AD=(Armagh* or Belfast* or Lisburn* or Londonderry* or Derry* or Newry*)

# 8 #1 OR #2 OR #3 OR #4 OR #5 OR #6 OR #7

**Key:**

TS= terms in either title, abstract, author keywords, and keywords plus fields

TI= terms in title field

AB= abstract field

AD= address field (which will find institution and place names)

CU= country/region in the address field

SO= publication titles field

OO= organization field

OG= affiliation field (previously called organization-enhanced)

## **The Adapted Filter Compared to the UK MEDLINE Filter:**

To view NICE’s UK MEDLINE filter please see: Ayiku L, Levay P, Hudson T, Craven J, Barrett E, Finnegan A, et al. The MEDLINE UK filter: development and validation of a geographic search filter to retrieve research about the UK from OVID MEDLINE. *Health Information Libraries Journal*. 2017;34(3):200-216. <http://dx.doi.org/10.1111/hir.12187>

| **Original Line Numbers** | **Original Line (Description)** | **Original Search Fields** | **Adapted to Lines** | **Adapted to (Description)** | **Adapted Search Fields** |
| --- | --- | --- | --- | --- | --- |
| 1 | Exploded medical subject heading for Great Britain | subject heading field | N/A | N/A | N/A |
| 2 | National Health Service terms | .ti,ab,in. | 1 | National Health Service terms | TS or (OO or OG or AD and AD) |
| 3 | English NOT irrelevant terms | .ti,ab. | 2 | English NOT irrelevant terms | TI or AB |
| 4 | UK country / nationality terms | .ti,ab,jw,in. | 3 | UK country / nationality terms | TS or SO or CU |
| 5 | English city terms | .ti,ab,in. | 4 | English city terms | TI or AB or AD |
| 6 | Welsh city terms | .ti,ab,in. | 5 | Welsh city terms | TI or AB or AD |
| 7 | Scottish city terms | .ti,ab,in. | 6 | Scottish city terms | TI or AB or AD |
| 8 | Northern Irish city terms | .ti,ab,in. | 7 | Northern Irish city terms | TI or AB or AD |
| 9 | Lines 1-8 OR-ed together | N/A | 8 | Lines 1-7 OR-ed together | N/A |
| 10 | Medical subject headings for continents NOT Great Britain or Europe | subject heading field | N/A | N/A | N/A |
| 11 | Line 9 NOT 10 | N/A | N/A | N/A | N/A |

**Original Filter (MEDLINE via Ovid) Key:**

MeSH **=** indexing term (Medical Subject Heading)

ti = title

ab = abstract

jw = journal word

in = institution

**Adapted Filter (Web of Science Core Collection) Key:**

TS= terms in either title, abstract, author keywords, and keywords plus fields

TI= terms in title field

AB= abstract field

AD= address field (which will find institution and place names)

CU= country/region in the address field

SO= publication titles field

OO= organization field

OG= affiliation field (previously called organization-enhanced)

**Changes Made to the Adapted Filter:**

• **Line 1** and **lines 10-11** of the NICE UK MEDLINE filter could not be adapted as there are no MeSH terms on the WoSCC databases.

• In **line 2** of the adapted filter, terms for the NHS were also searched in the author keywords and keyword plus fields.

• Terms for the NHS in the Organization, Affiliation or Address fields were AND-ed with UK country terms in the AD (address) field to reduce results from non-UK National Health Services (e.g., Italy).

• In **line 3** of the adapted filter, UK country/nationality terms and abbreviations were also searched in the author keywords and keyword plus fields.

• Terms in the publication field were adapted based on the results for the SO (publication titles) field using the ‘Analyze Results’ feature.

• The CU (countries/region) field was searched instead of the institution field. On this line, the terms "North Ireland*" and "North Irish*" were included to reflect how Northern Ireland is abbreviated on the WoSCC databases. Terms such as "G.B." and "U.K." were removed to reduce noise.

• In **line 4** of the adapted filter, city terms were included once with the truncation symbol on the end (instead of also searching for the city name with 's on the end, as in the NICE filter).

• The use of "" was removed except for in place names made up of 2 or more words (e.g. "New York*").

• Instead of the institution field, the AD (address) field was searched. Terms in the AD field were adapted – instead of removing irrelevant place names using NOT (and as in the TI and AB fields), terms were AND-ed with relevant place names. This was because the use of NOT could remove records where institutions collaborated with each other and because there were still records remaining for non-UK places. Although many (though not all) of the additional terms applied with AND had been searched elsewhere in the filter, country names were previously searched using the CU (countries/region) field, whereas the AND-ed terms were searched for in the AD (address) field which will find affiliations or place names within the address field of a record – this was tested and was found to reduce noise.

• In the AD (address) field, "St Alban*" was AND-ed with relevant place names as it was picking up results from non-UK St Albans. St Albans was also truncated as St Alban*.

• In **line 5** of the adapted filter, the same changes were made as in line 4. In addition, St David's was truncated to St David* and St Asaph's to St Asaph*.

• In **line 6** of the adapted filter, the same changes were made as in line 4.

• In **line 7** of the adapted filter, the same changes were made as in line 4.

# **Testing the Recall of the Inbuilt Limits and Search Filter on Datasets on/about the UK (Datasets 1-3):**

Below are all the individual searches performed when applying the limit and filters to the search strategies created to retain the records for each individual dataset (1-3 and 4). The initial strategy consisting of the title searches for datasets 1-3 is shown first. This section is not repeated in the sections that show each of the limits and the adapted filter when they are applied.

For two of the three datasets, relevance to a UK population was part of the screening criteria for the reviews, but no geographic limits or filters had been used (datasets 1 and 3). For the remaining dataset (dataset 2), relevance to OECD populations was part of the screening criteria for the review, with some non-OECD countries removed using the Boolean operator NOT in the search strategy. All the original searches were limited to English language publications.

For dataset 3, 203 included publications were in this dataset although the database each of the includes was retrieved on was not recorded in the data. We therefore tested each of the record titles on the WoSCC databases used in the review, Social Science Citation Index (SSCI) 1956-present, Conference Proceedings Citation Index – Social Science & Humanities (CPCI-SSH) 1990-present, and Emerging Sources Citation Index (ESCI) 2015-present, finding 61 records in total.

The Countries/Regions and Affiliations limits were only applied using the list of the top 200 record counts displayed.

## **Dataset 1:**

**Initial Strategy to Retrieve 6 Citations:**

**WoSCC Database:** Social Science Citation Index (SSCI) 1956-present

**Date searched:** 31st July 2023

**Records retrieved:** 6

1 TI=("reliability and use with international standards for body mass index") or TI=("Cause and effect beliefs and self-esteem of overweight children") or TI=("Children's stereotypes of overweight children") or TI=("Body shape perceptions of preadolescent and young adolescent children") or TI=("Eating habits, body-esteem and self-esteem in Scottish children and adolescents") or TI=("Mothers, daughters and dieting: investigating the transmission of weight control") 6

**Strategy Limited by Countries/Regions:**

**WoSCC Database:** Social Science Citation Index (SSCI) 1956-present

**Date searched:** 31st July 2023

**Records retrieved:** 5

2 and ENGLAND or SCOTLAND or WALES (Countries/Regions) 5

**Strategy Limited by Affiliations:**

**WoSCC Database:** Social Science Citation Index (SSCI) 1956-present

**Date searched:** 31st July 2023

**Records retrieved:** 5

2 and N8 RESEARCH PARTNERSHIP or CARDIFF UNIVERSITY or WHITE ROSE UNIVERSITY CONSORTIUM or UNIVERSITY OF LEEDS or UNIVERSITY OF LONDON or ROYAL HOSP SICK CHILDREN or ST GEORGES UNIVERSITY LONDON or NEWCASTLE UNIVERSITY UK or RLUK RESEARCH LIBRARIES UK (Affiliations) 5

**Strategy Limited by Filter:**

**WoSCC Database:** Social Science Citation Index (SSCI) 1956-present

**Date searched:** 31st July 2023

**Records retrieved:** 6

10 #9 AND #1 6

9 #8 OR #7 OR #6 OR #5 OR #4 OR #3 OR #2 2,086,259

8 TI=(Armagh* or Belfast* or Lisburn* or Londonderry* or Derry* or Newry*) or AB=(Armagh* or Belfast* or Lisburn* or Londonderry* or Derry* or Newry*) or AD=(Armagh* or Belfast* or Lisburn* or Londonderry* or Derry* or Newry*) 19,561

7 TI=(Aberdeen* or Dundee* or Edinburgh* or Glasgow* or Inverness or (Perth* not Australia*) or Stirling*) or AB=(Aberdeen* or Dundee* or Edinburgh* or Glasgow* or Inverness or (Perth* not Australia*) or Stirling*) or AD=(Aberdeen* or Dundee* or Edinburgh* or Glasgow* or Inverness or (Perth* and (Perthshire* or Scotland* or UK or "United Kingdom*")) or Stirling*) 118,443

6 TI=(Bangor* or Cardiff* or Newport* or "St Asaph*" or "St David*" or Swansea*) or AB=(Bangor* or Cardiff* or Newport* or "St Asaph*" or "St David*" or Swansea*) or AD=(Bangor* or Cardiff* or Newport* or "St Asaph*" or ("St David*" and (Pembrokeshire* or Wales* or UK or "United Kingdom*")) or Swansea*) 47,659

5 TI=(Bath* or (Birmingham* not Alabama*) or Bradford* or Brighton* or Bristol* or Carlisle* or (Cambridge* not (Massachusetts* or Boston* or Harvard*)) or (Canterbury* not Zealand*) or Chelmsford* or Chester* or Chichester* or Coventry* or Derby* or (Durham* not (Carolina* or NC)) or Ely* or Exeter* or Gloucester* or Hereford* or Hull* or Lancaster* or Leeds* or Leicester* or (Lincoln* not Nebraska*) or (Liverpool* not ("New South Wales*" or NSW)) or (London* not (Ontario* or ONT or Toronto*)) or Manchester* or (Newcastle* not ("New South Wales*" or NSW)) or Norwich* or Nottingham* or Oxford* or Peterborough* or Plymouth* or Portsmouth* or Preston* or Ripon* or Salford* or Salisbury* or Sheffield* or Southampton* or "St Alban*" or Stoke* or Sunderland* or Truro* or Wakefield* or Wells or Westminster* or Winchester* or Wolverhampton* or (Worcester* not (Massachusetts* or Boston* or Harvard*)) or (York* not ("New York*" or NY or Ontario* or ONT or Toronto*))) or AB=(Bath* or (Birmingham* not Alabama*) or Bradford* or Brighton* or Bristol* or Carlisle* or (Cambridge* not (Massachusetts* or Boston* or Harvard*)) or (Canterbury* not Zealand*) or Chelmsford* or Chester* or Chichester* or Coventry* or Derby* or (Durham* not (Carolina* or NC)) or Ely* or Exeter* or Gloucester* or Hereford* or Hull* or Lancaster* or Leeds* or Leicester* or (Lincoln* not Nebraska*) or (Liverpool* not ("New South Wales*" or NSW)) or (London* not (Ontario* or ONT or Toronto*)) or Manchester* or (Newcastle* not ("New South Wales*" or NSW)) or Norwich* or Nottingham* or Oxford* or Peterborough* or Plymouth* or Portsmouth* or Preston* or Ripon* or Salford* or Salisbury* or Sheffield* or Southampton* or "St Alban*" or Stoke* or Sunderland* or Truro* or Wakefield* or Wells or Westminster* or Winchester* or Wolverhampton* or (Worcester* not (Massachusetts* or Boston* or Harvard*)) or (York* not ("New York*" or NY or Ontario* or ONT or Toronto*))) or AD=(Bath* or (Birmingham* and (Midlands* or England* or UK or "United Kingdom*")) or Bradford* or Brighton* or Bristol* or Carlisle* or (Cambridge* and (Cambridgeshire* or England* or UK or "United Kingdom*")) or (Canterbury* and (Kent* or England* or UK or "United Kingdom*")) or Chelmsford* or Chester* or Chichester* or Coventry* or Derby* or (Durham* and ("County Durham*" or England* or UK or "United Kingdom*")) or Ely* or Exeter* or Gloucester* or Hereford* or Hull* or Lancaster* or Leeds* or Leicester* or (Lincoln* and (Lincolnshire* or England* or UK or "United Kingdom*")) or (Liverpool* and (Merseyside* or England* or UK or "United Kingdom*")) or (London* and (Borough* or Westminster* or "Kingston-upon-Thames" or Kensington* or Chelsea* or Greenwich* or England* or UK or "United Kingdom*")) or Manchester* or (Newcastle* and (Tyne* or Wear* or England* or UK or "United Kingdom*")) or Norwich* or Nottingham* or Oxford* or Peterborough* or Plymouth* or Portsmouth* or Preston* or Ripon* or Salford* or Salisbury* or Sheffield* or Southampton* or ("St Alban*" and (Hertfordshire* or England* or UK or "United Kingdom*")) or Stoke* or Sunderland* or Truro* or Wakefield* or Wells or Westminster* or Winchester* or Wolverhampton* or (Worcester* and (Worcestershire* or England* or UK or "United Kingdom*")) or (York* and (Yorkshire* or England* or UK or "United Kingdom*"))) 1,660,862

4 TS=(GB or Britain* or (British* not "British Columbia") or UK or "United Kingdom*" or (England* not "New England") or "Northern Ireland*" or "Northern Irish*" or "North Ireland*" or "North Irish*" or Scotland* or Scottish* or ((Wales or "South Wales") not "New South Wales") or Welsh*) or SO=(Britain* or British* or UK or "United Kingdom*" or England* or "Northern Ireland*" or "Northern Irish*" or Scotland* or Scottish* or Wales* or Welsh*) or CU=(Britain* or UK or "United Kingdom*" or England* or "North Ireland*" or "Northern Ireland*" or Scotland* or Wales*) 1,284,759

3 TI=(English not ((published or publication* or translat* or written or language* or speak* or literature or citation*) NEAR/5 English)) or AB=(English not ((published or publication* or translat* or written or language* or speak* or literature or citation*) NEAR/5 English)) 68,744

2 TS=("National Health Service*" or NHS*) or (OO=(National Health Service) or OG=(NHS*) or AD=(National Health Service or NHS*)) and (AD=(Britain* or UK or "United Kingdom*" or England* or "North Ireland*" or "Northern Ireland*" or Scotland* or Wales*)) 52,950

## **Dataset 2:**

**Initial Strategy to Retrieve 14 Records:**

**WoSCC Database:** Social Science Citation Index (SSCI) 1956-present

**Date searched:** 31st July 2023

**Records retrieved:** 14

1 TI=("qualitative investigation involving young people with cystic fibrosis") or TI=("Transition to adult mental health services for young people with attention deficit/hyperactivity disorder (ADHD)") or TI=(" Implementation of transitional care guidance in a UK paediatric and a neighbouring adult facility)") or TI=("a qualitative study of the experiences and suggestions of young people with diabetes") or TI=("balancing the views of young adults with intellectual disabilities and their carers in transition planning") or TI=("qualitative comparative embedded case study with young people with epilepsy and their parents during transition from children's to adult services") or TI=("adult services and adulthood for young people with life-limiting conditions and their families") or TI=("multiple perspectives on young people's experience of leaving care") or TI=("Pathways for young people with intellectual disabilities from out-of-area residential schools or colleges") or TI=("Transition in endocrinology: The challenge of maintaining continuity") or TI=("Transition for Teenagers With Intellectual Disability: Carers' Perspectives") or TI=("Team approach versus ad hoc health services for young people with physical disabilities: a retrospective cohort study") or TI=("Continuity of care in the transition from child to adult diabetes services: A realistic evaluation study") or TI=("the changing roles of parents in the transition from child to adult diabetes service") 14

**Strategy Limited by Countries/Regions:**

**WoSCC Database:** Social Science Citation Index (SSCI) 1956-present

**Date searched:** 31st July 2023

**Records retrieved:** 14

2 and ENGLAND or WALES (Countries/Regions) 14

**Strategy Limited by Affiliations:**

**WoSCC Database:** Social Science Citation Index (SSCI) 1956-present

**Date searched:** 31st July 2023

**Records retrieved:** 14

2 and N8 RESEARCH PARTNERSHIP or UNIVERSITY OF MANCHESTER or CARDIFF UNIVERSITY or CARDIFF VALE UNIV HLTH BOARD or UNIVERSITY OF BRISTOL or UNIVERSITY OF LEICESTER or UNIVERSITY OF NOTTINGHAM or BANGOR UNIVERSITY or LEICESTER CITY COUNCIL or LEICESTERSHIRE CTY COUNCIL or LEICESTERSHIRE PARTNERSHIP NHS TRUST or MANCHESTER ADULT CYST FIBROSIS CTR or NORTHUMBRIA HEALTHCARE NHS FDN TRUST or NOTTINGHAMSHIRE HEALTHCARE NHS TRUST or PLYMOUTH COMMUNITY HEALTHCARE CIC or ROYAL MANCHESTER CHILDREN S HOSPITAL or WHITE ROSE UNIVERSITY CONSORTIUM or UNIVERSITY OF YORK UK or UNIVERSITY OF SOUTH WALES or UNIVERSITY OF PLYMOUTH or UNIVERSITY OF LEEDS or UNIVERSITY OF BIRMINGHAM or SOMERSET PARTNERSHIP NHS FDN TRUST or SHEFFIELD CHILDREN S NHS FOUNDATION TRUST or SHEFFIELD CHILDREN S HOSPITAL (Affiliations) 14

**Strategy Limited by Filter:**

**WoSCC Database:** Social Science Citation Index (SSCI) 1956-present

**Date searched:** 31st July 2023

**Records retrieved:** 14

10 #9 AND #1 14

9 #8 OR #7 OR #6 OR #5 OR #4 OR #3 OR #2 2,086,259

8 TI=(Armagh* or Belfast* or Lisburn* or Londonderry* or Derry* or Newry*) or AB=(Armagh* or Belfast* or Lisburn* or Londonderry* or Derry* or Newry*) or AD=(Armagh* or Belfast* or Lisburn* or Londonderry* or Derry* or Newry*) 19,561

7 TI=(Aberdeen* or Dundee* or Edinburgh* or Glasgow* or Inverness or (Perth* not Australia*) or Stirling*) or AB=(Aberdeen* or Dundee* or Edinburgh* or Glasgow* or Inverness or (Perth* not Australia*) or Stirling*) or AD=(Aberdeen* or Dundee* or Edinburgh* or Glasgow* or Inverness or (Perth* and (Perthshire* or Scotland* or UK or "United Kingdom*")) or Stirling*) 118,443

6 TI=(Bangor* or Cardiff* or Newport* or "St Asaph*" or "St David*" or Swansea*) or AB=(Bangor* or Cardiff* or Newport* or "St Asaph*" or "St David*" or Swansea*) or AD=(Bangor* or Cardiff* or Newport* or "St Asaph*" or ("St David*" and (Pembrokeshire* or Wales* or UK or "United Kingdom*")) or Swansea*) 47,659

5 TI=(Bath* or (Birmingham* not Alabama*) or Bradford* or Brighton* or Bristol* or Carlisle* or (Cambridge* not (Massachusetts* or Boston* or Harvard*)) or (Canterbury* not Zealand*) or Chelmsford* or Chester* or Chichester* or Coventry* or Derby* or (Durham* not (Carolina* or NC)) or Ely* or Exeter* or Gloucester* or Hereford* or Hull* or Lancaster* or Leeds* or Leicester* or (Lincoln* not Nebraska*) or (Liverpool* not ("New South Wales*" or NSW)) or (London* not (Ontario* or ONT or Toronto*)) or Manchester* or (Newcastle* not ("New South Wales*" or NSW)) or Norwich* or Nottingham* or Oxford* or Peterborough* or Plymouth* or Portsmouth* or Preston* or Ripon* or Salford* or Salisbury* or Sheffield* or Southampton* or "St Alban*" or Stoke* or Sunderland* or Truro* or Wakefield* or Wells or Westminster* or Winchester* or Wolverhampton* or (Worcester* not (Massachusetts* or Boston* or Harvard*)) or (York* not ("New York*" or NY or Ontario* or ONT or Toronto*))) or AB=(Bath* or (Birmingham* not Alabama*) or Bradford* or Brighton* or Bristol* or Carlisle* or (Cambridge* not (Massachusetts* or Boston* or Harvard*)) or (Canterbury* not Zealand*) or Chelmsford* or Chester* or Chichester* or Coventry* or Derby* or (Durham* not (Carolina* or NC)) or Ely* or Exeter* or Gloucester* or Hereford* or Hull* or Lancaster* or Leeds* or Leicester* or (Lincoln* not Nebraska*) or (Liverpool* not ("New South Wales*" or NSW)) or (London* not (Ontario* or ONT or Toronto*)) or Manchester* or (Newcastle* not ("New South Wales*" or NSW)) or Norwich* or Nottingham* or Oxford* or Peterborough* or Plymouth* or Portsmouth* or Preston* or Ripon* or Salford* or Salisbury* or Sheffield* or Southampton* or "St Alban*" or Stoke* or Sunderland* or Truro* or Wakefield* or Wells or Westminster* or Winchester* or Wolverhampton* or (Worcester* not (Massachusetts* or Boston* or Harvard*)) or (York* not ("New York*" or NY or Ontario* or ONT or Toronto*))) or AD=(Bath* or (Birmingham* and (Midlands* or England* or UK or "United Kingdom*")) or Bradford* or Brighton* or Bristol* or Carlisle* or (Cambridge* and (Cambridgeshire* or England* or UK or "United Kingdom*")) or (Canterbury* and (Kent* or England* or UK or "United Kingdom*")) or Chelmsford* or Chester* or Chichester* or Coventry* or Derby* or (Durham* and ("County Durham*" or England* or UK or "United Kingdom*")) or Ely* or Exeter* or Gloucester* or Hereford* or Hull* or Lancaster* or Leeds* or Leicester* or (Lincoln* and (Lincolnshire* or England* or UK or "United Kingdom*")) or (Liverpool* and (Merseyside* or England* or UK or "United Kingdom*")) or (London* and (Borough* or Westminster* or "Kingston-upon-Thames" or Kensington* or Chelsea* or Greenwich* or England* or UK or "United Kingdom*")) or Manchester* or (Newcastle* and (Tyne* or Wear* or England* or UK or "United Kingdom*")) or Norwich* or Nottingham* or Oxford* or Peterborough* or Plymouth* or Portsmouth* or Preston* or Ripon* or Salford* or Salisbury* or Sheffield* or Southampton* or ("St Alban*" and (Hertfordshire* or England* or UK or "United Kingdom*")) or Stoke* or Sunderland* or Truro* or Wakefield* or Wells or Westminster* or Winchester* or Wolverhampton* or (Worcester* and (Worcestershire* or England* or UK or "United Kingdom*")) or (York* and (Yorkshire* or England* or UK or "United Kingdom*"))) 1,660,862

4 TS=(GB or Britain* or (British* not "British Columbia") or UK or "United Kingdom*" or (England* not "New England") or "Northern Ireland*" or "Northern Irish*" or "North Ireland*" or "North Irish*" or Scotland* or Scottish* or ((Wales or "South Wales") not "New South Wales") or Welsh*) or SO=(Britain* or British* or UK or "United Kingdom*" or England* or "Northern Ireland*" or "Northern Irish*" or Scotland* or Scottish* or Wales* or Welsh*) or CU=(Britain* or UK or "United Kingdom*" or England* or "North Ireland*" or "Northern Ireland*" or Scotland* or Wales*) 1,284,759

3 TI=(English not ((published or publication* or translat* or written or language* or speak* or literature or citation*) NEAR/5 English)) or AB=(English not ((published or publication* or translat* or written or language* or speak* or literature or citation*) NEAR/5 English)) 68,744

2 TS=("National Health Service*" or NHS*) or (OO=(National Health Service) or OG=(NHS*) or AD=(National Health Service or NHS*)) and (AD=(Britain* or UK or "United Kingdom*" or England* or "North Ireland*" or "Northern Ireland*" or Scotland* or Wales*)) 52,950

## **Dataset 3:**

**Initial Strategy to Retrieve 61 Records:**

**WoSCC Databases:** Social Science Citation Index (SSCI) 1956-present, Conference Proceedings Citation Index – Social Science & Humanities (CPCI-SSH) 1990-present, and Emerging Sources Citation Index (ESCI) 2015-present

**Date searched:** 1st August 2023

**Records retrieved:** 61

1 TI=("Identification of patients with atrial fibrillation in UK community pharmacy: an evaluation of a new service") or TI=("Pharmacy counter assistants and oral health promotion: an exploratory study") or TI=("Comparison of range of commercial or primary care led weight reduction programmes with minimal intervention control for weight loss in obesity: lighten Up randomised controlled trial") or TI=("Why people seek advice from community pharmacies about skin problems") or TI=("Views of English Pharmacists on Providing Public Health Services") or TI=("Using the eSexual Health Clinic to access chlamydia treatment and care via the internet: a qualitative interview study") or TI=("Understanding public trust in services provided by community pharmacists relative to those provided by general practitioners: a qualitative study") or TI=("The role of the community pharmacist in drug abuse: a comparison of service provision between Northern Ireland and England/Wales") or TI=("The role of community pharmacists in supporting self-management in patients with psoriasis") or TI=("An exploration of attitudes to extended roles for community pharmacists") or TI=("The public's perception of the role of community pharmacists in Wales") or TI=("The provision of current and future sexual health services from community pharmacies in Grampian, Scotland") or TI=("The impact of training and delivering alcohol brief intervention on the knowledge and attitudes of community pharmacists: a before and after study") or TI=("goal-focused intervention for people with intermittent allergic rhinitis") or TI=("The frequency of anxiety and depression in purchasers of over-the-counter sleep aids") or TI=("The feasibility and acceptability of the provision of alcohol screening and brief advice in pharmacies for women accessing emergency contraception: an evaluation study") or TI=("The effectiveness of brief alcohol interventions delivered by community pharmacists: randomized controlled trial") or TI=("The effect of an intervention programme to improve health education leaflet uptake and distribution in community pharmacies") or TI=("The current and future roles of community pharmacists: views and experiences of patients with type 2 diabetes") or TI=("The case for targeting community pharmacy-led health improvement: Findings from a skin cancer campaign in Wales") or TI=("Support for community pharmacy-based alcohol interventions: a Scottish general public survey") or TI=("Strategies enhancing the public health role of community pharmacists: a qualitative study") or TI=("'Standing Outside the Junkie Door'-service users' experiences of using community pharmacies to access treatment for opioid dependency") or TI=("Self-care of long-term conditions: patients' perspectives and their (limited) use of community pharmacies") or TI=("Restructuring supervision and reconfiguration of skill mix in community pharmacy: Classification of perceived safety and risk") or TI=("Quality improvement of community pharmacy services: a prioritisation exercise") or TI=("Provision of emergency hormonal contraception through community pharmacies in a rural area") or TI=("Providing dental health services to drug users: testing a model for a community pharmacy advice and referral scheme") or TI=("Preferences of community pharmacists for extended roles in primary care: a survey and discrete choice experiment") or TI=("Pharmacy needle exchange: do clients and community pharmacists have matching perceptions") or TI=("Pharmaceutical care of older people: what do older people want from community pharmacy") or TI=("Meeting the health needs of problematic drug users through community pharmacy: a qualitative study") or TI=("Involving the public and other stakeholders in development and evaluation of a community pharmacy alcohol screening and brief advice service") or TI=("Improving uptake of the copper intrauterine device for emergency contraception by educating pharmacists in the community") or TI=("How to enhance public health service utilization in community") or TI=("How do community pharmacists conceptualise and operationalise self-care support of long-term conditions (LTCs)? An English cross-sectional survey") or TI=("Helpful advice and hidden expertize: pharmacy users' experiences of community pharmacy accessibility") or TI=("Health professionals' attitudes to the deregulation of emergency contraception (or the problem of female sexuality)") or TI=("General public's views on pharmacy public health services: current situation and opportunities in the future") or TI=("From community pharmacy to healthy living pharmacy: positive early experiences from Portsmouth, England") or TI=("Evaluation of the Healthy LifeCheck programme: a vascular risk assessment service for community pharmacies in Leicester city, UK") or TI=("Evaluation of NHS Health Checks provided by community pharmacies") or TI=("Evaluation of a young person's sexual health service in a commercial setting") or TI=("Evaluation of a community pharmacy delivered oral contraception service") or TI=("Evaluation of a cardiovascular disease opportunistic risk assessment pilot ('Heart MOT' service) in community pharmacies") or TI=("England's Healthy Living Pharmacy (HLP) initiative: Facilitating the engagement of pharmacy support staff in public health") or TI=("DOT-C: A cluster randomised feasibility trial evaluating directly observed anti-HCV therapy in a population receiving opioid substitute therapy from community pharmacy")or TI=("Does recovery-oriented treatment prompt heroin users prematurely into detoxification and abstinence") or TI=("Coronary heart disease: health knowledge and behaviour") or TI=("Contested space in the pharmacy: public attitudes to pharmacy harm reduction services in the West of Scotland") or TI=("Community pharmacy service users' views and perceptions of alcohol screening and brief intervention") or TI=("Community pharmacies and the provision of opioid substitution services for drug misusers: changes in activity and attitudes of community pharmacists across England 1995-2005") or TI=("Benefits and tensions in delivering public health in community pharmacies - a qualitative study of healthy living pharmacy staff champions") or TI=("An exploratory study of the views of community pharmacy staff on the management of patients with undiagnosed skin problems") or TI=("A survey of the quality and accuracy of information leaflets about skin cancer and sun-protective behaviour available from UK general practices and community pharmacies") or TI=("A survey of community pharmacists on prevention of HIV and hepatitis B and C: current practice and attitudes in Grampian") or TI=("A quasi-experimental evaluation of dried blood spot testing through community pharmacies in the Tayside region of Scotland") or TI=("A qualitative study of English community pharmacists' experiences of providing lifestyle advice to patients with cardiovascular disease") or TI=("A drop-in clinic for patients with poorly-controlled diabetes: a community pharmacy feasibility study") or TI=("A survey to identify barriers in the public health role of community pharmacists") and AD=(Middlesex) or TI=("Over-the-counter advice seeking about complementary and alternative medicines (CAM) in community pharmacies and health shops: an ethnographic study") 61

**Strategy Limited by Countries/Regions:**

**WoSCC Databases:** Social Science Citation Index (SSCI) 1956-present, Conference Proceedings Citation Index – Social Science & Humanities (CPCI-SSH) 1990-present, and Emerging Sources Citation Index (ESCI) 2015-present

**Date searched:** 1st August 2023

**Records retrieved:** 59

2 and ENGLAND or SCOTLAND or WALES or NORTH IRELAND (Countries/Regions) 59

**Strategy Limited by Affiliations:**

**WoSCC Databases:** Social Science Citation Index (SSCI) 1956-present, Conference Proceedings Citation Index – Social Science & Humanities (CPCI-SSH) 1990-present, and Emerging Sources Citation Index (ESCI) 2015-present

**Date searched:** 1st August 2023

**Records retrieved:** 58

2 and UNIVERSITY OF LONDON or N8 RESEARCH PARTNERSHIP or KING S COLLEGE LONDON or UNIVERSITY OF ABERDEEN or ROBERT GORDON UNIVERSITY or UNIVERSITY COLLEGE LONDON or UNIVERSITY OF DUNDEE or LIVERPOOL JOHN MOORES UNIVERSITY or RLUK RESEARCH LIBRARIES UK or UNIVERSITY OF GREENWICH or UNIVERSITY OF KENT or UNIVERSITY OF MANCHESTER or UNIVERSITY OF STIRLING or DURHAM UNIVERSITY or UNIVERSITY OF PORTSMOUTH or UNIVERSITY OF EAST ANGLIA or UNIVERSITY OF GLASGOW or UNIVERSITY OF LIVERPOOL or UNIVERSITY OF STRATHCLYDE or UNIVERSITY OF YORK UK or ARBROATH INFIRM or ASTON UNIVERSITY or BANGOR UNIVERSITY or BRUNEL UNIVERSITY or CARDIFF METROPOLITAN UNIVERSITY or CITY UNIVERSITY LONDON or DURHAM CTY COUNCIL or GLASGOW CALEDONIAN UNIVERSITY or GREATER GLASGOW HLTH BOARD or GUY S ST THOMAS NHS FOUNDATION TRUST or HAMBLETON RICHMONDSHIRE PCT or KEELE UNIVERSITY or KENSINGTON CHELSEA WESTMINSTER HLTH AUTHOR or UNIVERSITY OF BRISTOL or UNIVERSITY OF BIRMINGHAM or ST GEORGES UNIVERSITY LONDON or SOUTH LONDON MAUDSLEY NHS TRUST or QUEENS UNIVERSITY BELFAST or QUEEN MARY UNIVERSITY LONDON or MIDDLESEX UNIVERSITY or LIVERPOOL SCHOOL OF TROPICAL MEDICINE or CARDIFF UNIVERSITY or WHITE ROSE UNIVERSITY CONSORTIUM or LEICESTER GENERAL HOSPITAL or LONDON SCHOOL OF HYGIENE TROPICAL MEDICINE or NHS S BIRMINGHAM or NHS SHEFFIELD CLIN COMMISSIONING GRP or NHS TAYSIDE or OXFORD BROOKES UNIVERSITY or PUBL HLTH WALES or PUBLIC HEALTH ENGLAND or UNIVERSITY OF BATH or UNIVERSITY OF BRIGHTON or UNIVERSITY OF CAMBRIDGE or UNIVERSITY OF EDINBURGH or UNIVERSITY OF LONDON SCHOOL OF PHARMACY or UNIVERSITY OF PLYMOUTH or UNIVERSITY OF SHEFFIELD or UNIVERSITY OF SOUTHAMPTON or UNIVERSITY OF SUSSEX or UNIVERSITY OF SUNDERLAND or UNIVERSITY OF WALES TRINITY ST DAVID or UNIVERSITY OF WOLVERHAMPTON or UNIVERSITY HOSPITALS OF LEICESTER NHS TRUST or SOUTHWARK PRIMARY CARE TRUST or NURSING MIDWIFERY COUNCIL (Affiliations) 58

**Strategy Limited by Filter:**

**WoSCC Databases:** Social Science Citation Index (SSCI) 1956-present, Conference Proceedings Citation Index – Social Science & Humanities (CPCI-SSH) 1990-present, and Emerging Sources Citation Index (ESCI) 2015-present

**Date searched:** 1st August 2023

**Records retrieved:** 61

10 #9 AND #1 61

9 #8 OR #7 OR #6 OR #5 OR #4 OR #3 OR #2 2,876,094

8 TI=(Armagh* or Belfast* or Lisburn* or Londonderry* or Derry* or Newry*) or AB=(Armagh* or Belfast* or Lisburn* or Londonderry* or Derry* or Newry*) or AD=(Armagh* or Belfast* or Lisburn* or Londonderry* or Derry* or Newry*) 23,470

7 TI=(Aberdeen* or Dundee* or Edinburgh* or Glasgow* or Inverness or (Perth* not Australia*) or Stirling*) or AB=(Aberdeen* or Dundee* or Edinburgh* or Glasgow* or Inverness or (Perth* not Australia*) or Stirling*) or AD=(Aberdeen* or Dundee* or Edinburgh* or Glasgow* or Inverness or (Perth* and (Perthshire* or Scotland* or UK or "United Kingdom*")) or Stirling*) 144,469

6 TI=(Bangor* or Cardiff* or Newport* or "St Asaph*" or "St David*" or Swansea*) or AB=(Bangor* or Cardiff* or Newport* or "St Asaph*" or "St David*" or Swansea*) or AD=(Bangor* or Cardiff* or Newport* or "St Asaph*" or ("St David*" and (Pembrokeshire* or Wales* or UK or "United Kingdom*")) or Swansea*) 55,997

5 TI=(Bath* or (Birmingham* not Alabama*) or Bradford* or Brighton* or Bristol* or Carlisle* or (Cambridge* not (Massachusetts* or Boston* or Harvard*)) or (Canterbury* not Zealand*) or Chelmsford* or Chester* or Chichester* or Coventry* or Derby* or (Durham* not (Carolina* or NC)) or Ely* or Exeter* or Gloucester* or Hereford* or Hull* or Lancaster* or Leeds* or Leicester* or (Lincoln* not Nebraska*) or (Liverpool* not ("New South Wales*" or NSW)) or (London* not (Ontario* or ONT or Toronto*)) or Manchester* or (Newcastle* not ("New South Wales*" or NSW)) or Norwich* or Nottingham* or Oxford* or Peterborough* or Plymouth* or Portsmouth* or Preston* or Ripon* or Salford* or Salisbury* or Sheffield* or Southampton* or "St Alban*" or Stoke* or Sunderland* or Truro* or Wakefield* or Wells or Westminster* or Winchester* or Wolverhampton* or (Worcester* not (Massachusetts* or Boston* or Harvard*)) or (York* not ("New York*" or NY or Ontario* or ONT or Toronto*))) or AB=(Bath* or (Birmingham* not Alabama*) or Bradford* or Brighton* or Bristol* or Carlisle* or (Cambridge* not (Massachusetts* or Boston* or Harvard*)) or (Canterbury* not Zealand*) or Chelmsford* or Chester* or Chichester* or Coventry* or Derby* or (Durham* not (Carolina* or NC)) or Ely* or Exeter* or Gloucester* or Hereford* or Hull* or Lancaster* or Leeds* or Leicester* or (Lincoln* not Nebraska*) or (Liverpool* not ("New South Wales*" or NSW)) or (London* not (Ontario* or ONT or Toronto*)) or Manchester* or (Newcastle* not ("New South Wales*" or NSW)) or Norwich* or Nottingham* or Oxford* or Peterborough* or Plymouth* or Portsmouth* or Preston* or Ripon* or Salford* or Salisbury* or Sheffield* or Southampton* or "St Alban*" or Stoke* or Sunderland* or Truro* or Wakefield* or Wells or Westminster* or Winchester* or Wolverhampton* or (Worcester* not (Massachusetts* or Boston* or Harvard*)) or (York* not ("New York*" or NY or Ontario* or ONT or Toronto*))) or AD=(Bath* or (Birmingham* and (Midlands* or England* or UK or "United Kingdom*")) or Bradford* or Brighton* or Bristol* or Carlisle* or (Cambridge* and (Cambridgeshire* or England* or UK or "United Kingdom*")) or (Canterbury* and (Kent* or England* or UK or "United Kingdom*")) or Chelmsford* or Chester* or Chichester* or Coventry* or Derby* or (Durham* and ("County Durham*" or England* or UK or "United Kingdom*")) or Ely* or Exeter* or Gloucester* or Hereford* or Hull* or Lancaster* or Leeds* or Leicester* or (Lincoln* and (Lincolnshire* or England* or UK or "United Kingdom*")) or (Liverpool* and (Merseyside* or England* or UK or "United Kingdom*")) or (London* and (Borough* or Westminster* or "Kingston-upon-Thames" or Kensington* or Chelsea* or Greenwich* or England* or UK or "United Kingdom*")) or Manchester* or (Newcastle* and (Tyne* or Wear* or England* or UK or "United Kingdom*")) or Norwich* or Nottingham* or Oxford* or Peterborough* or Plymouth* or Portsmouth* or Preston* or Ripon* or Salford* or Salisbury* or Sheffield* or Southampton* or ("St Alban*" and (Hertfordshire* or England* or UK or "United Kingdom*")) or Stoke* or Sunderland* or Truro* or Wakefield* or Wells or Westminster* or Winchester* or Wolverhampton* or (Worcester* and (Worcestershire* or England* or UK or "United Kingdom*")) or (York* and (Yorkshire* or England* or UK or "United Kingdom*"))) 2,347,230

4 TS=(GB or Britain* or (British* not "British Columbia") or UK or "United Kingdom*" or (England* not "New England") or "Northern Ireland*" or "Northern Irish*" or "North Ireland*" or "North Irish*" or Scotland* or Scottish* or ((Wales or "South Wales") not "New South Wales") or Welsh*) or SO=(Britain* or British* or UK or "United Kingdom*" or England* or "Northern Ireland*" or "Northern Irish*" or Scotland* or Scottish* or Wales* or Welsh*) or CU=(Britain* or UK or "United Kingdom*" or England* or "North Ireland*" or "Northern Ireland*" or Scotland* or Wales*) 1,534,850

3 TI=(English not ((published or publication* or translat* or written or language* or speak* or literature or citation*) NEAR/5 English)) or AB=(English not ((published or publication* or translat* or written or language* or speak* or literature or citation*) NEAR/5 English)) 103,871

2 TS=("National Health Service*" or NHS*) or (OO=(National Health Service) or OG=(NHS*) or AD=(National Health Service or NHS*)) and (AD=(Britain* or UK or "United Kingdom*" or England* or "North Ireland*" or "Northern Ireland*" or Scotland* or Wales*)) 74,542

## **Dataset 4:**

Below are all the individual searches performed when applying the limit and the adapted filter to the DOI search strategy created to retain the records in dataset 4. The initial strategy consists of the DOIs.

One DOI ("10.1136/bmj.d3952") was removed from the strategy, as it was found not be on or about a UK setting.

The Countries/Regions and Affiliations limits were only applied using the list of the top 200 record counts displayed.

**Initial Strategy to Retrieve 96 Records:**

**WoSCC Databases:** Science Citation Index Expanded (SCI-Expanded) 1900-present; Social Sciences Citation Index (SSCI) 1956-present; Arts & Humanities Citation Index (AHCI) 1975-present; Conference Proceedings Citation Index – Science (CPCI-S) 1990-present; Conference Proceedings Citation Index – Social Science & Humanities (CPCI-SSH) 1990-present; and Emerging Sources Citation Index (ESCI) 2015-present

**Date searched:** 1st August 2023

**Records retrieved:** 96

1 DO=("10.1136/jclinpath-2012-201056" OR "10.1016/j.bjps.2012.06.017" OR "10.1111/j.1360-0443.2012.03955.x" OR "10.3310/hta16260" OR "10.7326/0003-4819-156-6-201203200-00003" OR "10.1093/eurheartj/ehs017" OR "10.1056/NEJMoa1110556" OR "10.1093/ntr/ntr260" OR "10.1016/j.jvs.2011.08.040" OR "10.1002/ibd.21938" OR "10.1002/ijc.26394" OR "10.1016/j.numecd.2010.09.010" OR "10.1111/j.1475-3588.2010.00587.x" OR "10.1016/j.crohns.2009.02.005" OR "10.1016/j.crohns.2008.10.004" OR "10.1586/14737167.4.1.27" OR "10.1056/NEJMoa1103799" OR "10.1016/j.jaac.2011.09.017" OR "10.1186/1471-2458-11-803" OR "10.1016/j.ijom.2011.04.020" OR "10.1111/j.1526-4637.2011.01215.x" OR "10.1093/eurheartj/ehr340" OR "10.1056/NEJMoa1107039" OR "10.1007/s00330-011-2226-z" OR "10.2337/dc11-0606" OR "10.1038/bjc.2011.202" OR "10.1186/1471-2458-11-370" OR "10.1111/j.1360-0443.2011.03505.x" OR "10.1093/ntr/ntr030" OR "10.1016/j.ejcts.2011.02.027" OR "10.1136/bjsm.2010.082503" OR "10.1002/jbmr.364" OR "10.1016/j.jaac.2010.12.015" OR "10.1016/j.rbmo.2010.11.005" OR "10.1056/NEJMoa1007432" OR "10.1111/j.1751-7893.2010.00254.x" OR "10.1136/adc.2010.183111" OR "10.1371/journal.pmed.1000386" OR "10.1007/s00787-010-0148-y" OR "10.1136/adc.2010.183129" OR "10.1093/humrep/deq339" OR "10.1053/j.ajkd.2010.08.037" OR "10.1136/hrt.2010.198382" OR "10.1177/0333102410381142" OR "10.1016/j.bjps.2010.04.039" OR "10.1093/ndt/gfq219" OR "10.1016/S1470-2045(10)70263-1" OR "10.1016/j.brat.2010.07.011" OR "10.1111/j.1469-7610.2010.02302.x" OR "10.1148/radiol.10100094" OR "10.1093/humrep/deq213" OR "10.3109/14647273.2010.495763" OR "10.1186/1471-2458-10-463" OR "10.3310/hta14380" OR "10.1016/j.jvs.2010.04.077" OR "10.1080/15374416.2010.486315" OR "10.1016/j.socscimed.2010.04.031" OR "10.1001/archfacial.2010.26" OR "10.1056/NEJMoa0909545" OR "10.1177/0020764008101855" OR "10.3111/13696990903562861" OR "10.1111/j.1464-410X.2009.09160.x" OR "10.1371/journal.pone.0008933" OR "10.1177/0272989X09349961" OR "10.1111/j.1469-7610.2009.02127.x" OR "10.1016/j.pec.2009.04.004" OR "10.1016/j.genhosppsych.2009.08.003" OR "10.1159/000235916" OR "10.1192/bjp.bp.108.056531" OR "10.1136/emj.2008.061598" OR "10.1056/NEJMoa0900563" OR "10.1016/j.rmed.2009.04.025" OR "10.1136/adc.2009.159095" OR "10.1185/03007990903116255" OR "10.1016/j.ygyno.2009.06.012" OR "10.1111/j.1365-2214.2009.00975.x" OR "10.3310/hta13270" OR "10.1016/j.fas.2008.08.005" OR "10.1016/j.brat.2009.02.009" OR "10.3174/ajnr.A1519" OR "10.1016/S0140-6736(09)60010-6" OR "10.1093/ageing/afn281" OR "10.1136/adc.2008.145011" OR "10.1136/thx.2007.091249" OR "10.1093/ndt/gfn488" OR "10.1007/s11255-008-9448-2" OR "10.1111/j.1365-2036.2008.03856.x" OR "10.1111/j.1742-1241.2008.01879.x" OR "10.1136/bmj.a716" OR "10.1159/000145462" OR "10.1016/j.rmed.2008.06.002" OR "10.1111/j.1464-5491.2008.02476.x" OR "10.1185/03007990802234829" OR "10.1053/j.ajkd.2008.03.020" OR "10.1093/ageing/afn062" OR "10.1111/j.1463-1326.2008.00886.x" OR "10.1161/CIRCULATIONAHA.107.735779" OR "10.1111/j.1464-5491.2008.02430.x" OR "10.1093/eurheartj/ehn073" OR "10.1007/s11136-008-9311-z" OR "10.1023/A:1025624221369" OR "10.1023/A:1025611818643" OR "10.1016/0304-3959(85)90024-7") 96

**Strategy Limited by Countries/Regions:**

**WoSCC Databases:** Science Citation Index Expanded (SCI-Expanded) 1900-present; Social Sciences Citation Index (SSCI) 1956-present; Arts & Humanities Citation Index (AHCI) 1975-present; Conference Proceedings Citation Index – Science (CPCI-S) 1990-present; Conference Proceedings Citation Index – Social Science & Humanities (CPCI-SSH) 1990-present; and Emerging Sources Citation Index (ESCI) 2015-present

**Date searched:** 1st August 2023

**Records retrieved:** 88

2 and ENGLAND or SCOTLAND or WALES or NORTH IRELAND (Countries/Regions) 88

**Strategy Limited by Affiliations:**

**WoSCC Databases:** Science Citation Index Expanded (SCI-Expanded) 1900-present; Social Sciences Citation Index (SSCI) 1956-present; Arts & Humanities Citation Index (AHCI) 1975-present; Conference Proceedings Citation Index – Science (CPCI-S) 1990-present; Conference Proceedings Citation Index – Social Science & Humanities (CPCI-SSH) 1990-present; and Emerging Sources Citation Index (ESCI) 2015-present

**Date searched:** 1st August 2023

**Records retrieved:** 81

2 and N8 RESEARCH PARTNERSHIP or UNIVERSITY OF LONDON or UNIVERSITY COLLEGE LONDON or UNIVERSITY OF OXFORD or IMPERIAL COLLEGE LONDON or UNIVERSITY OF MANCHESTER or WHITE ROSE UNIVERSITY CONSORTIUM or UNIVERSITY OF BIRMINGHAM or UNIVERSITY OF LIVERPOOL or UNIVERSITY OF NOTTINGHAM or CARDIFF UNIVERSITY or KING S COLLEGE LONDON or UNIVERSITY OF ABERDEEN or UNIVERSITY OF EDINBURGH or UNIVERSITY OF GLASGOW or UNIVERSITY OF SHEFFIELD or BANGOR UNIVERSITY or GREAT ORMOND STREET HOSPITAL FOR CHILDREN NHS FOUNDATION TRUST or NOTTINGHAM UNIVERSITY HOSPITAL NHS TRUST or UNIVERSITY OF BRISTOL or GUY S ST THOMAS NHS FOUNDATION TRUST or NOTTINGHAM CITY HOSPITAL or RLUK RESEARCH LIBRARIES UK or UNIVERSITY COLLEGE LONDON HOSPITALS NHS FOUNDATION TRUST or UNIVERSITY OF CAMBRIDGE or AINTREE UNIVERSITY HOSPITALS NHS FOUNDATION TRUST or CANCER RESEARCH UK or CHRISTIE NHS FOUNDATION TRUST or HEART OF ENGLAND NHS FOUNDATION TRUST or KING S COLLEGE HOSPITAL or KEMRI WELLCOME TRUST RES PROGRAMME or KING S COLLEGE HOSPITAL NHS FOUNDATION TRUST or LONDON SCHOOL ECONOMICS POLITICAL SCIENCE or LOUGHBOROUGH UNIVERSITY or NEWCASTLE UNIVERSITY UK or QUEEN MARY UNIVERSITY LONDON or UNIVERSITY OF LEEDS or UNIVERSITY OF LEICESTER or UNIVERSITY OF WARWICK or BARTS HEALTH NHS TRUST or BRUNEL UNIVERSITY or CENT MANCHESTER FDN TRUST or CHELSEA WESTMINSTER ASSISTED CONCEPT UNIT or GLASGOW CALEDONIAN UNIVERSITY or HEART HOSP NHS TRUST or INSTITUTE OF CANCER RESEARCH UK or KINGSTON HOSP NHS TRUST or KLEIJNEN SYSTEMATIC REVIEWS or LONDON SCHOOL OF HYGIENE TROPICAL MEDICINE or LIVERPOOL HEART CHEST HOSPITAL or BIRMINGHAM CITY UNIVERSITY or BIRMINGHAM SOLIHULL MENTAL HLTH FDN TRUST or BARNSLEY DIST GEN HOSP or BRISTOL ROYAL INFIRMARY or BUPA HLTH WELLBEING or DEPT HLTH or FALKIRK DIST ROYAL INFIRM or UNIVERSITY OF DUNDEE or ROYAL COLL PAEDIAT CHILD HLTH RCPCH (Affiliations) 81

**Strategy Limited by Filter:**

**WoSCC Database:** Science Citation Index Expanded (SCI-Expanded) 1900-present; Social Sciences Citation Index (SSCI) 1956-present; Arts & Humanities Citation Index (AHCI) 1975-present; Conference Proceedings Citation Index – Science (CPCI-S) 1990-present; Conference Proceedings Citation Index – Social Science & Humanities (CPCI-SSH) 1990-present; and Emerging Sources Citation Index (ESCI) 2015-present

**Date searched:** 1st August 2023

**Records retrieved:** 91

10 #9 AND #1 91

9 #8 OR #7 OR #6 OR #5 OR #4 OR #3 OR #2 14,311,304

8 TI=(Armagh* or Belfast* or Lisburn* or Londonderry* or Derry* or Newry*) or AB=(Armagh* or Belfast* or Lisburn* or Londonderry* or Derry* or Newry*) or AD=(Armagh* or Belfast* or Lisburn* or Londonderry* or Derry* or Newry*) 118,116

7 TI=(Aberdeen* or Dundee* or Edinburgh* or Glasgow* or Inverness or (Perth* not Australia*) or Stirling*) or AB=(Aberdeen* or Dundee* or Edinburgh* or Glasgow* or Inverness or (Perth* not Australia*) or Stirling*) or AD=(Aberdeen* or Dundee* or Edinburgh* or Glasgow* or Inverness or (Perth* and (Perthshire* or Scotland* or UK or "United Kingdom*")) or Stirling*) 752,251

6 TI=(Bangor* or Cardiff* or Newport* or "St Asaph*" or "St David*" or Swansea*) or AB=(Bangor* or Cardiff* or Newport* or "St Asaph*" or "St David*" or Swansea*) or AD=(Bangor* or Cardiff* or Newport* or "St Asaph*" or ("St David*" and (Pembrokeshire* or Wales* or UK or "United Kingdom*")) or Swansea*) 240,793

5 TI=(Bath* or (Birmingham* not Alabama*) or Bradford* or Brighton* or Bristol* or Carlisle* or (Cambridge* not (Massachusetts* or Boston* or Harvard*)) or (Canterbury* not Zealand*) or Chelmsford* or Chester* or Chichester* or Coventry* or Derby* or (Durham* not (Carolina* or NC)) or Ely* or Exeter* or Gloucester* or Hereford* or Hull* or Lancaster* or Leeds* or Leicester* or (Lincoln* not Nebraska*) or (Liverpool* not ("New South Wales*" or NSW)) or (London* not (Ontario* or ONT or Toronto*)) or Manchester* or (Newcastle* not ("New South Wales*" or NSW)) or Norwich* or Nottingham* or Oxford* or Peterborough* or Plymouth* or Portsmouth* or Preston* or Ripon* or Salford* or Salisbury* or Sheffield* or Southampton* or "St Alban*" or Stoke* or Sunderland* or Truro* or Wakefield* or Wells or Westminster* or Winchester* or Wolverhampton* or (Worcester* not (Massachusetts* or Boston* or Harvard*)) or (York* not ("New York*" or NY or Ontario* or ONT or Toronto*))) or AB=(Bath* or (Birmingham* not Alabama*) or Bradford* or Brighton* or Bristol* or Carlisle* or (Cambridge* not (Massachusetts* or Boston* or Harvard*)) or (Canterbury* not Zealand*) or Chelmsford* or Chester* or Chichester* or Coventry* or Derby* or (Durham* not (Carolina* or NC)) or Ely* or Exeter* or Gloucester* or Hereford* or Hull* or Lancaster* or Leeds* or Leicester* or (Lincoln* not Nebraska*) or (Liverpool* not ("New South Wales*" or NSW)) or (London* not (Ontario* or ONT or Toronto*)) or Manchester* or (Newcastle* not ("New South Wales*" or NSW)) or Norwich* or Nottingham* or Oxford* or Peterborough* or Plymouth* or Portsmouth* or Preston* or Ripon* or Salford* or Salisbury* or Sheffield* or Southampton* or "St Alban*" or Stoke* or Sunderland* or Truro* or Wakefield* or Wells or Westminster* or Winchester* or Wolverhampton* or (Worcester* not (Massachusetts* or Boston* or Harvard*)) or (York* not ("New York*" or NY or Ontario* or ONT or Toronto*))) or AD=(Bath* or (Birmingham* and (Midlands* or England* or UK or "United Kingdom*")) or Bradford* or Brighton* or Bristol* or Carlisle* or (Cambridge* and (Cambridgeshire* or England* or UK or "United Kingdom*")) or (Canterbury* and (Kent* or England* or UK or "United Kingdom*")) or Chelmsford* or Chester* or Chichester* or Coventry* or Derby* or (Durham* and ("County Durham*" or England* or UK or "United Kingdom*")) or Ely* or Exeter* or Gloucester* or Hereford* or Hull* or Lancaster* or Leeds* or Leicester* or (Lincoln* and (Lincolnshire* or England* or UK or "United Kingdom*")) or (Liverpool* and (Merseyside* or England* or UK or "United Kingdom*")) or (London* and (Borough* or Westminster* or "Kingston-upon-Thames" or Kensington* or Chelsea* or Greenwich* or England* or UK or "United Kingdom*")) or Manchester* or (Newcastle* and (Tyne* or Wear* or England* or UK or "United Kingdom*")) or Norwich* or Nottingham* or Oxford* or Peterborough* or Plymouth* or Portsmouth* or Preston* or Ripon* or Salford* or Salisbury* or Sheffield* or Southampton* or ("St Alban*" and (Hertfordshire* or England* or UK or "United Kingdom*")) or Stoke* or Sunderland* or Truro* or Wakefield* or Wells or Westminster* or Winchester* or Wolverhampton* or (Worcester* and (Worcestershire* or England* or UK or "United Kingdom*")) or (York* and (Yorkshire* or England* or UK or "United Kingdom*"))) 11,918,475

4 TS=(GB or Britain* or (British* not "British Columbia") or UK or "United Kingdom*" or (England* not "New England") or "Northern Ireland*" or "Northern Irish*" or "North Ireland*" or "North Irish*" or Scotland* or Scottish* or ((Wales or "South Wales") not "New South Wales") or Welsh*) or SO=(Britain* or British* or UK or "United Kingdom*" or England* or "Northern Ireland*" or "Northern Irish*" or Scotland* or Scottish* or Wales* or Welsh*) or CU=(Britain* or UK or "United Kingdom*" or England* or "North Ireland*" or "Northern Ireland*" or Scotland* or Wales*) 6,950,305

3 TI=(English not ((published or publication* or translat* or written or language* or speak* or literature or citation*) NEAR/5 English)) or AB=(English not ((published or publication* or translat* or written or language* or speak* or literature or citation*) NEAR/5 English)) 189,028

2 TS=("National Health Service*" or NHS*) or (OO=(National Health Service) or OG=(NHS*) or AD=(National Health Service or NHS*)) and (AD=(Britain* or UK or "United Kingdom*" or England* or "North Ireland*" or "Northern Ireland*" or Scotland* or Wales*)) 344,477

# **Determining the Reduction in Overall Number-Needed-to-Screen offered by the Inbuilt Limits Versus the Adapted Search Filter:**

Below are the reproduction searches for datasets 1 and 3, which are shown individually. The limits and the adapted filter were applied to each reproduction search in one continuous strategy. Line numbers noting the application of either of the limits or filter are listed.

For these datasets, relevance to a UK population was part of the screening criteria for the original reviews, but no geographic limits or filters had been used. The endeavour to reduce ONNS was a theoretical exercise had the UK been the sole geographic focus of each review.

The Countries/Regions and Affiliations limits were only applied using the list of the top 200 record counts displayed.

## **Dataset 1:**

**WoSCC Database:** Social Science Citation Index (SSCI) 1956-present

**Date searched:** 1^st^ August 2023

The search strategy was originally performed on 6 June 2008. An index date of 1900-01-01 to 2008-06-06 was applied to every line to replicate the strategy as closely as possible. Exact search was turned off for all lines; hits were refined by document types: Articles or Meeting Abstracts; limited to English language; and to publication years 1997-2008.

1,915 records were retrieved by the original search, whereas 2,416 were retrieved by the attempt to replicate the original search.

- Lines 1-7 show the reproduction search.
- Lines 8-15 show the translated filter. The filter is applied at line 16.
- Line 17 shows the application of the Countries/Regions limit.
- Line 18 shows the application of the Affiliations limit.

18 #3 AND #2 AND #1 and Article or Meeting Abstract (Document Types) and English (Languages) and 1997 or 1998 or 2000 or 1999 or 2001 or 2002 or 2003 or 2004 or 2005 or 2006 or 2007 or 2008 (Publication Years) and KING S COLLEGE LONDON or LEEDS BECKETT UNIVERSITY or LOUGHBOROUGH UNIVERSITY or N8 RESEARCH PARTNERSHIP or RLUK RESEARCH LIBRARIES UK or UNIVERSITY COLLEGE LONDON or UNIVERSITY OF BRISTOL or UNIVERSITY OF CAMBRIDGE or UNIVERSITY OF EDINBURGH or UNIVERSITY OF GLASGOW or UNIVERSITY OF LEEDS or UNIVERSITY OF LIVERPOOL or UNIVERSITY OF LONDON or UNIVERSITY OF OXFORD or WHITE ROSE UNIVERSITY CONSORTIUM (Affiliations) 168

17 #3 AND #2 AND #1 and Article or Meeting Abstract (Document Types) and English (Languages) and 1997 or 1998 or 2000 or 1999 or 2001 or 2002 or 2003 or 2004 or 2005 or 2006 or 2007 or 2008 (Publication Years) and ENGLAND or NORTH IRELAND or SCOTLAND or WALES (Countries/Regions) 261

16 #15 AND #7 631

15 #8 OR #9 OR #10 OR #11 OR #12 OR #13 OR #14 817,537

14 TI=(Armagh* or Belfast* or Lisburn* or Londonderry* or Derry* or Newry*) or AB=(Armagh* or Belfast* or Lisburn* or Londonderry* or Derry* or Newry*) or AD=(Armagh* or Belfast* or Lisburn* or Londonderry* or Derry* or Newry*) 8,113

13 TI=(Aberdeen* or Dundee* or Edinburgh* or Glasgow* or Inverness or (Perth* not Australia*) or Stirling*) or AB=(Aberdeen* or Dundee* or Edinburgh* or Glasgow* or Inverness or (Perth* not Australia*) or Stirling*) or AD=(Aberdeen* or Dundee* or Edinburgh* or Glasgow* or Inverness or (Perth* and (Perthshire* or Scotland* or UK or "United Kingdom*")) or Stirling*) 49,695

12 TI=(Bangor* or Cardiff* or Newport* or "St Asaph*" or "St David*" or Swansea*) or AB=(Bangor* or Cardiff* or Newport* or "St Asaph*" or "St David*" or Swansea*) or AD=(Bangor* or Cardiff* or Newport* or "St Asaph*" or ("St David*" and (Pembrokeshire* or Wales* or UK or "United Kingdom*")) or Swansea*) 20,367

11 TI=(Bath* or (Birmingham* not Alabama*) or Bradford* or Brighton* or Bristol* or Carlisle* or (Cambridge* not (Massachusetts* or Boston* or Harvard*)) or (Canterbury* not Zealand*) or Chelmsford* or Chester* or Chichester* or Coventry* or Derby* or (Durham* not (Carolina* or NC)) or Ely* or Exeter* or Gloucester* or Hereford* or Hull* or Lancaster* or Leeds* or Leicester* or (Lincoln* not Nebraska*) or (Liverpool* not ("New South Wales*" or NSW)) or (London* not (Ontario* or ONT or Toronto*)) or Manchester* or (Newcastle* not ("New South Wales*" or NSW)) or Norwich* or Nottingham* or Oxford* or Peterborough* or Plymouth* or Portsmouth* or Preston* or Ripon* or Salford* or Salisbury* or Sheffield* or Southampton* or "St Alban*" or Stoke* or Sunderland* or Truro* or Wakefield* or Wells or Westminster* or Winchester* or Wolverhampton* or (Worcester* not (Massachusetts* or Boston* or Harvard*)) or (York* not ("New York*" or NY or Ontario* or ONT or Toronto*))) or AB=(Bath* or (Birmingham* not Alabama*) or Bradford* or Brighton* or Bristol* or Carlisle* or (Cambridge* not (Massachusetts* or Boston* or Harvard*)) or (Canterbury* not Zealand*) or Chelmsford* or Chester* or Chichester* or Coventry* or Derby* or (Durham* not (Carolina* or NC)) or Ely* or Exeter* or Gloucester* or Hereford* or Hull* or Lancaster* or Leeds* or Leicester* or (Lincoln* not Nebraska*) or (Liverpool* not ("New South Wales*" or NSW)) or (London* not (Ontario* or ONT or Toronto*)) or Manchester* or (Newcastle* not ("New South Wales*" or NSW)) or Norwich* or Nottingham* or Oxford* or Peterborough* or Plymouth* or Portsmouth* or Preston* or Ripon* or Salford* or Salisbury* or Sheffield* or Southampton* or "St Alban*" or Stoke* or Sunderland* or Truro* or Wakefield* or Wells or Westminster* or Winchester* or Wolverhampton* or (Worcester* not (Massachusetts* or Boston* or Harvard*)) or (York* not ("New York*" or NY or Ontario* or ONT or Toronto*))) or AD=(Bath* or (Birmingham* and (Midlands* or England* or UK or "United Kingdom*")) or Bradford* or Brighton* or Bristol* or Carlisle* or (Cambridge* and (Cambridgeshire* or England* or UK or "United Kingdom*")) or (Canterbury* and (Kent* or England* or UK or "United Kingdom*")) or Chelmsford* or Chester* or Chichester* or Coventry* or Derby* or (Durham* and ("County Durham*" or England* or UK or "United Kingdom*")) or Ely* or Exeter* or Gloucester* or Hereford* or Hull* or Lancaster* or Leeds* or Leicester* or (Lincoln* and (Lincolnshire* or England* or UK or "United Kingdom*")) or (Liverpool* and (Merseyside* or England* or UK or "United Kingdom*")) or (London* and (Borough* or Westminster* or "Kingston-upon-Thames" or Kensington* or Chelsea* or Greenwich* or England* or UK or "United Kingdom*")) or Manchester* or (Newcastle* and (Tyne* or Wear* or England* or UK or "United Kingdom*")) or Norwich* or Nottingham* or Oxford* or Peterborough* or Plymouth* or Portsmouth* or Preston* or Ripon* or Salford* or Salisbury* or Sheffield* or Southampton* or ("St Alban*" and (Hertfordshire* or England* or UK or "United Kingdom*")) or Stoke* or Sunderland* or Truro* or Wakefield* or Wells or Westminster* or Winchester* or Wolverhampton* or (Worcester* and (Worcestershire* or England* or UK or "United Kingdom*")) or (York* and (Yorkshire* or England* or UK or "United Kingdom*"))) 581,871

10 TS=(GB or Britain* or (British* not "British Columbia") or UK or "United Kingdom*" or (England* not "New England") or "Northern Ireland*" or "Northern Irish*" or "North Ireland*" or "North Irish*" or Scotland* or Scottish* or ((Wales or "South Wales") not "New South Wales") or Welsh*) or SO=(Britain* or British* or UK or "United Kingdom*" or England* or "Northern Ireland*" or "Northern Irish*" or Scotland* or Scottish* or Wales* or Welsh*) or CU=(Britain* or UK or "United Kingdom*" or England* or "North Ireland*" or "Northern Ireland*" or Scotland* or Wales*) 611,706

9 TI=(English not ((published or publication* or translat* or written or language* or speak* or literature or citation*) NEAR/5 English)) or AB=(English not ((published or publication* or translat* or written or language* or speak* or literature or citation*) NEAR/5 English)) 25,127

8 TS=("National Health Service*" or NHS*) or (OO=(National Health Service) or OG=(NHS*) or AD=(National Health Service or NHS*)) and (AD=(Britain* or UK or "United Kingdom*" or England* or "North Ireland*" or "Northern Ireland*" or Scotland* or Wales*)) 9,873

7 #3 AND #2 AND #1 and Article or Meeting Abstract (Document Types) and English (Languages) and 1997 or 1998 or 2000 or 1999 or 2001 or 2002 or 2003 or 2004 or 2005 or 2006 or 2007 or 2008 (Publication Years) 2,416

6 #3 AND #2 AND #1 and Article or Meeting Abstract (Document Types) and English (Languages) 2,722

5 #3 AND #2 AND #1 and Article or Meeting Abstract (Document Types) 2,846

4 #3 AND #2 AND #1 3,042

3 (TS=(understanding OR understand OR opinion OR opinions OR concept OR attitude OR interview OR interviews OR interviewer OR interviewing OR interviewed) or TS=(in depth) OR TS=(case study) OR TS=(qualitative) OR TS=(Qualitative Research) OR TS=(Questionnaires) OR TS=(Questionnaire) OR TS=(self concept) OR TS=(comprehension) OR TS=(awareness) OR TS=(emotions) OR TS=(view OR views OR perception OR perceptions OR perceive OR perceives OR perceived OR attitudes OR attitudinal OR attitude OR belief OR beliefs OR concept OR concepts OR idea OR ideas OR perspectives OR perspective OR meaning OR meanings OR dissatisfaction) OR TS=(behavioral research) OR TS=(ethnology OR narration OR interviews) OR TS=(focus groups) OR TS=(ethnopsychology)) OR TS=(life experiences or human science) OR TS=(discourse* analysis) OR TS=(narrative analysis) OR TS=(lived experience*) OR TS=(field research) OR TS=(field studies) OR TS=(field study) OR TS=(focus group*) OR TS=(purpos* sampl*) OR TS=(constant comparison) OR TS=(constant comparative) OR TS=(grounded research) OR TS=(grounded studies) OR TS=(grounded study) OR TS=(grounded theor*) 918,514

2 TS=(body size) or TS=(body weight) or TS=(overweight OR thinness OR obese OR obesity OR obesogenic) or TS=(body weights) or TS=(body shape) or TS=(body SAME weight) or TS=(body SAME fat) 25,288

1 (TI=(boys OR boy OR girl OR girls) or TS=(child OR children OR childs OR childrens OR schoolchild OR schoolchildren OR "young boys" OR "young boy" OR "young girl" OR "young girls" OR preadolescent OR prepubescent OR kid OR kids)) 267,199

## **Dataset 3:**

**WoSCC Databases:** Social Science Citation Index (SSCI) 1956-present, Conference Proceedings Citation Index – Social Science & Humanities (CPCI-SSH) 1990-present, and Emerging Sources Citation Index (ESCI) 2015-present

**Date searched:** 1st August 2023

The search strategy was originally performed on 29 November 2017. An index date of 1956-01-01 to 2017-11-29 was applied to every line. Exact search was turned off for all lines, and records were limited to English language and publication years 2000-2017. 5,284 records were retrieved by the original search, whereas 5,377 were retrieved by the reproduced search.

- Lines 1-21 show the reproduction search.
- Lines 22-29 show the adapted filter. The filter is applied at line 30.
- Line 31 shows the application of the Countries/Regions limit.
- Line 32 shows the application of the Affiliations limit.

32 #18 OR #10 and English (Languages) and 2000 or 2001 or 2002 or 2003 or 2004 or 2005 or 2006 or 2007 or 2008 or 2009 or 2010 or 2011 or 2012 or 2013 or 2014 or 2015 or 2016 or 2017 (Publication Years) and ASTON UNIVERSITY or CARDIFF UNIVERSITY or IMPERIAL COLLEGE LONDON or LIVERPOOL JOHN MOORES UNIVERSITY or KING S COLLEGE LONDON or LONDON SCHOOL OF HYGIENE TROPICAL MEDICINE or N8 RESEARCH PARTNERSHIP or QUEENS UNIVERSITY BELFAST or RLUK RESEARCH LIBRARIES UK or ROBERT GORDON UNIVERSITY or UNIVERSITY COLLEGE LONDON or UNIVERSITY OF ABERDEEN or UNIVERSITY OF BIRMINGHAM or UNIVERSITY OF BRISTOL or UNIVERSITY OF EAST ANGLIA or UNIVERSITY OF GREENWICH or UNIVERSITY OF LEEDS or UNIVERSITY OF LONDON or UNIVERSITY OF LONDON SCHOOL OF PHARMACY or UNIVERSITY OF MANCHESTER or UNIVERSITY OF NOTTINGHAM or WHITE ROSE UNIVERSITY CONSORTIUM (Affiliations) 505

31 #18 OR #10 and English (Languages) and 2000 or 2001 or 2002 or 2003 or 2004 or 2005 or 2006 or 2007 or 2008 or 2009 or 2010 or 2011 or 2012 or 2013 or 2014 or 2015 or 2016 or 2017 (Publication Years) and ENGLAND or SCOTLAND or WALES or NORTH IRELAND (Countries/Regions) 698

30 #29 AND #21 1,560

29 #28 OR #27 OR #26 OR #25 OR #24 OR #23 OR #22 1,715,798

28 TI=(Armagh* or Belfast* or Lisburn* or Londonderry* or Derry* or Newry*) or AB=(Armagh* or Belfast* or Lisburn* or Londonderry* or Derry* or Newry*) or AD=(Armagh* or Belfast* or Lisburn* or Londonderry* or Derry* or Newry*) 15,186

27 TI=(Aberdeen* or Dundee* or Edinburgh* or Glasgow* or Inverness or (Perth* not Australia*) or Stirling*) or AB=(Aberdeen* or Dundee* or Edinburgh* or Glasgow* or Inverness or (Perth* not Australia*) or Stirling*) or AD=(Aberdeen* or Dundee* or Edinburgh* or Glasgow* or Inverness or (Perth* and (Perthshire* or Scotland* or UK or "United Kingdom*")) or Stirling*) 93,964

26 TI=(Bangor* or Cardiff* or Newport* or "St Asaph*" or "St David*" or Swansea*) or AB=(Bangor* or Cardiff* or Newport* or "St Asaph*" or "St David*" or Swansea*) or AD=(Bangor* or Cardiff* or Newport* or "St Asaph*" or ("St David*" and (Pembrokeshire* or Wales* or UK or "United Kingdom*")) or Swansea*) 38,018

25 TI=(Bath* or (Birmingham* not Alabama*) or Bradford* or Brighton* or Bristol* or Carlisle* or (Cambridge* not (Massachusetts* or Boston* or Harvard*)) or (Canterbury* not Zealand*) or Chelmsford* or Chester* or Chichester* or Coventry* or Derby* or (Durham* not (Carolina* or NC)) or Ely* or Exeter* or Gloucester* or Hereford* or Hull* or Lancaster* or Leeds* or Leicester* or (Lincoln* not Nebraska*) or (Liverpool* not ("New South Wales*" or NSW)) or (London* not (Ontario* or ONT or Toronto*)) or Manchester* or (Newcastle* not ("New South Wales*" or NSW)) or Norwich* or Nottingham* or Oxford* or Peterborough* or Plymouth* or Portsmouth* or Preston* or Ripon* or Salford* or Salisbury* or Sheffield* or Southampton* or "St Alban*" or Stoke* or Sunderland* or Truro* or Wakefield* or Wells or Westminster* or Winchester* or Wolverhampton* or (Worcester* not (Massachusetts* or Boston* or Harvard*)) or (York* not ("New York*" or NY or Ontario* or ONT or Toronto*))) or AB=(Bath* or (Birmingham* not Alabama*) or Bradford* or Brighton* or Bristol* or Carlisle* or (Cambridge* not (Massachusetts* or Boston* or Harvard*)) or (Canterbury* not Zealand*) or Chelmsford* or Chester* or Chichester* or Coventry* or Derby* or (Durham* not (Carolina* or NC)) or Ely* or Exeter* or Gloucester* or Hereford* or Hull* or Lancaster* or Leeds* or Leicester* or (Lincoln* not Nebraska*) or (Liverpool* not ("New South Wales*" or NSW)) or (London* not (Ontario* or ONT or Toronto*)) or Manchester* or (Newcastle* not ("New South Wales*" or NSW)) or Norwich* or Nottingham* or Oxford* or Peterborough* or Plymouth* or Portsmouth* or Preston* or Ripon* or Salford* or Salisbury* or Sheffield* or Southampton* or "St Alban*" or Stoke* or Sunderland* or Truro* or Wakefield* or Wells or Westminster* or Winchester* or Wolverhampton* or (Worcester* not (Massachusetts* or Boston* or Harvard*)) or (York* not ("New York*" or NY or Ontario* or ONT or Toronto*))) or AD=(Bath* or (Birmingham* and (Midlands* or England* or UK or "United Kingdom*")) or Bradford* or Brighton* or Bristol* or Carlisle* or (Cambridge* and (Cambridgeshire* or England* or UK or "United Kingdom*")) or (Canterbury* and (Kent* or England* or UK or "United Kingdom*")) or Chelmsford* or Chester* or Chichester* or Coventry* or Derby* or (Durham* and ("County Durham*" or England* or UK or "United Kingdom*")) or Ely* or Exeter* or Gloucester* or Hereford* or Hull* or Lancaster* or Leeds* or Leicester* or (Lincoln* and (Lincolnshire* or England* or UK or "United Kingdom*")) or (Liverpool* and (Merseyside* or England* or UK or "United Kingdom*")) or (London* and (Borough* or Westminster* or "Kingston-upon-Thames" or Kensington* or Chelsea* or Greenwich* or England* or UK or "United Kingdom*")) or Manchester* or (Newcastle* and (Tyne* or Wear* or England* or UK or "United Kingdom*")) or Norwich* or Nottingham* or Oxford* or Peterborough* or Plymouth* or Portsmouth* or Preston* or Ripon* or Salford* or Salisbury* or Sheffield* or Southampton* or ("St Alban*" and (Hertfordshire* or England* or UK or "United Kingdom*")) or Stoke* or Sunderland* or Truro* or Wakefield* or Wells or Westminster* or Winchester* or Wolverhampton* or (Worcester* and (Worcestershire* or England* or UK or "United Kingdom*")) or (York* and (Yorkshire* or England* or UK or "United Kingdom*"))) 1,334,141

24 TS=(GB or Britain* or (British* not "British Columbia") or UK or "United Kingdom*" or (England* not "New England") or "Northern Ireland*" or "Northern Irish*" or "North Ireland*" or "North Irish*" or Scotland* or Scottish* or ((Wales or "South Wales") not "New South Wales") or Welsh*) or SO=(Britain* or British* or UK or "United Kingdom*" or England* or "Northern Ireland*" or "Northern Irish*" or Scotland* or Scottish* or Wales* or Welsh*) or CU=(Britain* or UK or "United Kingdom*" or England* or "North Ireland*" or "Northern Ireland*" or Scotland* or Wales*) 1,064,078

23 TI=(English not ((published or publication* or translat* or written or language* or speak* or literature or citation*) NEAR/5 English)) or AB=(English not ((published or publication* or translat* or written or language* or speak* or literature or citation*) NEAR/5 English)) 61,042

22 TS=("National Health Service*" or NHS*) or (OO=(National Health Service) or OG=(NHS*) or AD=(National Health Service or NHS*)) and (AD=(Britain* or UK or "United Kingdom*" or England* or "North Ireland*" or "Northern Ireland*" or Scotland* or Wales*)) 34,808

21 #18 OR #10 and English (Languages) and 2000 or 2001 or 2002 or 2003 or 2004 or 2005 or 2006 or 2007 or 2008 or 2009 or 2010 or 2011 or 2012 or 2013 or 2014 or 2015 or 2016 or 2017 (Publication Years) 5,377

20 #18 OR #10 5,963

19 #18 NOT #5 111

18 #17 AND #1 278

17 #11 OR #12 OR #13 OR #14 OR #15 OR #16 115,400

16 ((TS=(alcoholic or alcoholics or alcoholism))) 28,866

15 ((TS=((drinking or alcohol*) NEAR/2 (abus* or misuse* or excess* or binge or problem* or risk* or habit* or control or manag* or reduc* or improve*)))) 37,538

14 (((TS=((alcohol) NEAR/3 (advice or consult* or counsel* or coach* or mentor* or assess* or issues or conversation* or enquir* or inquir* or intervention* or program* or project or model* or scheme* or initiative* or service* or group or groups or class or classes or club or clubs or camp or camps or consum* or educat* or biofeedback or support* or test* OR monitor* OR assess* OR screen* OR information OR program* OR access* OR communicat* OR refer OR referral*))))) 32,308

13 (((TS=((drinking) NEAR/3 (advice or consult* or counsel* or coach* or mentor* or assess* or issues or conversation* or enquir* or inquir* or intervention* or program* or project or model* or scheme* or initiative* or service* or group or groups or class or classes or club or clubs or camp or camps or consum* or educat* or biofeedback or support* or test* OR monitor* OR assess* OR screen* OR information OR program* OR access* OR communicat* OR refer OR referral*))))) 8,697

12 ((TS=((weight or obesity) NEAR/3 (advice or consult* or counsel* or coach* or mentor* or assess* or issues or conversation* or enquir* or inquir* or intervention* or program* or project or model* or scheme* or initiative* or service* or group or groups or class or classes or club or clubs or camp or camps or consum* or educat* or biofeedback or support* or test* OR monitor* OR assess* OR screen* OR information OR program* OR access* OR communicat* OR refer OR referral*)))) 24,319

11 ((TS=(((reduc* or decreas* or treat* or manag* or control* or improv*) NEAR/6 (obes* or "weight gain" or "weight loss" or overweight or "over weight" or "under weight" or underweight)) or (weight NEAR/1 (manag* or reduc* or control* or maintain* or improv* or treat* or decreas*))))) 21,257

10 #7 NOT #8 and English (Languages) 5,842

9 #7 NOT #8 6,176

8 TI=((developing NEAR/2 countr*) OR (developing NEAR/2 nation?)) OR TI=(Brasil or Brazil or Malaysia OR Thailand OR India OR China OR Nigeria OR Pakistan OR Tanzania OR Kenya) 204,184

7 #5 NOT #6 6,490

6 TI=(diagnose* OR hospital*) NOT TI=(community OR communities) 76,139

5 #4 AND #1 6,731

4 #3 OR #2 692,136

3 TS=( ("Health literacy" OR "health education" OR "health promotion" OR "preventive health" OR "primary prevention" OR "health information" OR (expanded NEAR/2 service?) OR "healthy living" OR "healthful living" OR "lifestyle" OR "life style" OR "public health" OR "population health" OR "promoting health" OR "health promoting" OR "health promotion" OR "health maintenance" OR "secondary prevention" OR vaccination? OR immunisation? OR immunization? OR screening OR "emergency contracept*")) 271,751

2 TS=( (pharmacy NEAR/2 led) OR (pharmacy NEAR/2 managed) OR (pharmacy NEAR/2 service?) OR (pharmacies NEAR/2 led) OR (pharmacies NEAR/2 managed) OR (pharmacies NEAR/2 service?) OR (pharmacist* NEAR/2 led) OR (pharmacist* NEAR/2 managed) OR (pharmacist* NEAR/2 service?) ) OR TS= ((community NEAR/10 (pharmacy OR pharmacies OR pharmacist*))) OR TS= ("community setting" OR "community member?") OR TS=(community NEAR/2 service?) OR TS=(community NEAR/2 (screen* OR test* OR promot* OR prevent*) ) OR TI=(pharmacy AND (screen* OR test* OR promot* OR prevent*) ) OR TI=(pharmacies AND (screen* OR test* OR promot* OR prevent*) ) OR TS=(pharmacy NEAR/3 (screen* OR test* OR promot* OR prevent*) ) OR TS=(pharmacies NEAR/3 (screen* OR test* OR promot* OR prevent*)) OR TI=((pharmacist* OR druggist* OR chemist? OR chemist's) AND (screen* OR test* OR promot* OR prevent* OR delivered)) OR TS=((pharmacist* OR druggist* OR chemist? OR chemist's) NEAR/2 (screen* OR test* OR promot* OR prevent* OR delivered)) OR TS=( (pharmacies NEAR/2 (screen* OR test* OR promot* OR prevent*) ) ) OR TS=("Lay pharmacist" OR "volunteer pharmacist" OR "Lay pharmacists" OR "volunteer pharmacists" OR "non dispensing" OR "Lay pharmacy" OR "volunteer pharmacy" OR "Lay pharmacies" OR "volunteer pharmacies" OR Drugstore OR "Drug store" OR Drugstores OR "Drug stores" OR "chain stores" OR "pharmacy shops" OR "pharmacy stores" OR "chemist shop" OR "chemist shops" OR "chemist store" OR "chemist stores" OR "high street chemist" or communities OR "local area" OR "local service" OR "local services" OR "local pharmacy" OR "local pharmacist" OR "local pharmacies" OR "local pharmacists" or locality or "high street" OR "high streets" OR shopping OR "high street" OR "high streets" OR precinct OR precincts OR parade OR parades OR shops OR mall OR malls OR supermarket OR supermarkets OR "chain pharmacy" OR "chain pharmacies" OR "retail pharmacy" OR "local population" OR "local populations" OR "general population" OR "geographic location" OR "general public" OR "population level" OR neighbourhood OR neighbourhoods OR neighborhood OR neighborhoods) OR TI=((pharmacy OR pharmacies OR pharmacist*) AND (city or cities or village? OR town?)) OR TS=((pharmacy OR pharmacies OR pharmacist*) NEAR/5 (city or cities or village? OR town?)) 474,272

1 TS=((Drugstore OR "Drug store" OR Drugstores OR "Drug stores" OR Pharmacy OR pharmacies OR pharmacists OR druggist OR druggists OR chemist's OR "chemist shop" OR "chemist shops" OR "chemist store" OR "chemist stores" OR "high street chemist?")) 16,328
